# Analysis on gene modular network reveals morphogen-directed development robustness in *Drosophila*

**DOI:** 10.1038/s41421-020-0173-z

**Published:** 2020-06-30

**Authors:** Shuo Zhang, Juan Zhao, Xiangdong Lv, Jialin Fan, Yi Lu, Tao Zeng, Hailong Wu, Luonan Chen, Yun Zhao

**Affiliations:** 1 State Key Laboratory of Cell Biology, Shanghai Institute of Biochemistry and Cell Biology, Center for Excellence in Molecular Cell Science, Chinese Academy of Sciences, University of Chinese Academy of Sciences, 200031 Shanghai, China; 2 University of Chinese Academy of Sciences, 100049 Beijing, China; 3 CAS Center for Excellence in Animal Evolution and Genetics, Chinese Academy of Sciences, Kunming, 650223 Yunnan China; 4 School of Life Science and Technology, ShanghaiTech University, 201210 Shanghai, China; 5 Key Laboratory of Systems Biology, Hangzhou Institute for Advanced Study, University of Chinese Academy of Sciences, Chinese Academy of Sciences, Hangzhou, 310024 Zhejiang China; 6 School of Life Science, Hangzhou Institute for Advanced Study, University of Chinese Academy of Sciences, Hangzhou, 310024 Zhejiang China

**Keywords:** Developmental biology, Bioinformatics

## Abstract

Genetic robustness is an important characteristic to tolerate genetic or nongenetic perturbations and ensure phenotypic stability. Morphogens, a type of evolutionarily conserved diffusible molecules, govern tissue patterns in a direction-dependent or concentration-dependent manner by differentially regulating downstream gene expression. However, whether the morphogen-directed gene regulatory network possesses genetic robustness remains elusive. In the present study, we collected 4217 morphogen-responsive genes along A-P axis of *Drosophila* wing discs from the RNA-seq data, and clustered them into 12 modules. By applying mathematical model to the measured data, we constructed a gene modular network (GMN) to decipher the module regulatory interactions and robustness in morphogen-directed development. The computational analyses on asymptotical dynamics of this GMN demonstrated that this morphogen-directed GMN is robust to tolerate a majority of genetic perturbations, which has been further validated by biological experiments. Furthermore, besides the genetic alterations, we further demonstrated that this morphogen-directed GMN can well tolerate nongenetic perturbations (Hh production changes) via computational analyses and experimental validation. Therefore, these findings clearly indicate that the morphogen-directed GMN is robust in response to perturbations and is important for *Drosophila* to ensure the proper tissue patterning in wing disc.

## Introduction

All of the multicellular organisms display specific body patterns, which is a result of the systemic incorporation of individual tissues or organs. Therefore, correct tissue patterns are essential for body pattern formation. Tissue patterns are well organized in early development process in both vertebrates and invertebrates, and are mainly determined by the morphogen gradients, which could further subdivide the tissue into different regions to fulfill the specific function^1–3^.

Morphogens are evolutionarily conserved, diffusible and long-range signaling molecules that govern the tissue pattern formation by regulating the expression of downstream genes in a distance-dependent and/or concentration-dependent manner^1–4^. Because of the diffusible property, different concentrations of morphogens thus form the morphogen gradient, which results in differential gene expression profiles carrying tissue positional information^5–10^. The morphogen gradient plays an indispensable role in tissue patterning and is widely investigated in the mouse neural tube, the limb bud, the blastoderm, the anterior–posterior (A–P) and dorsal–ventral (D–V) axes of *Drosophila* imaginal discs, especially in the wing imaginal disc^2,7,11^.

**Fig. 1:**
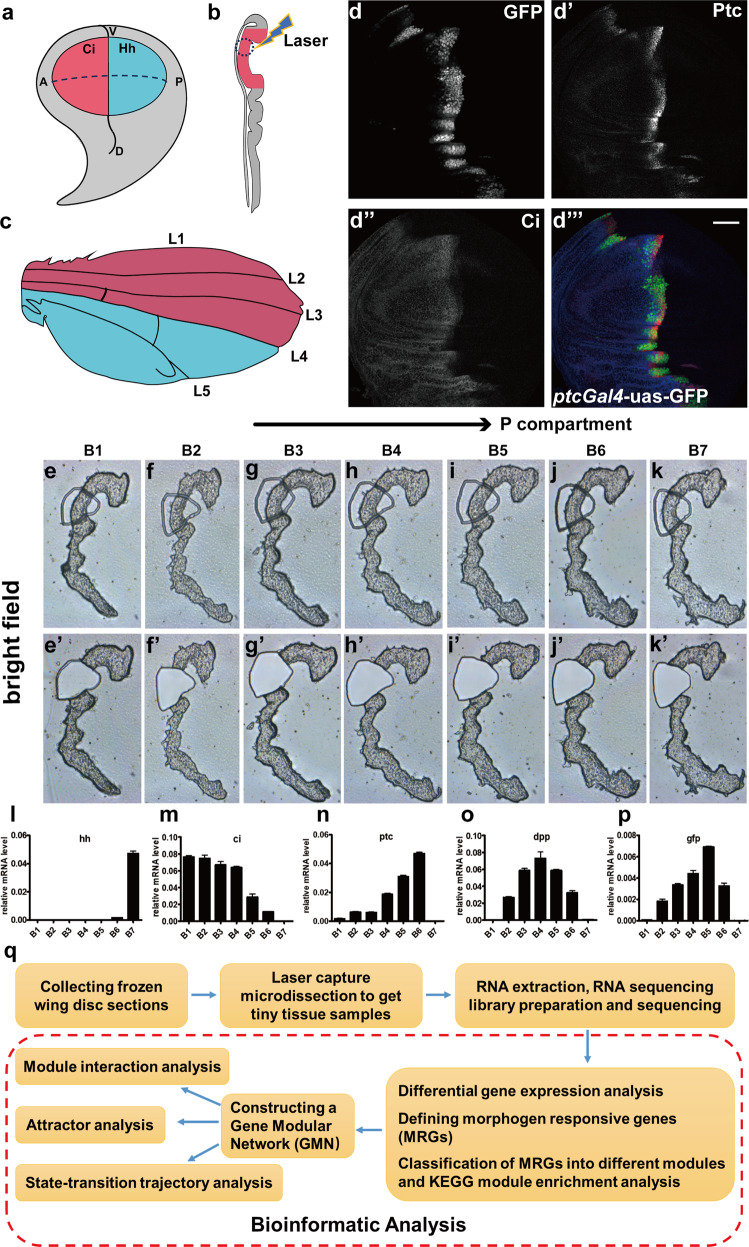
In vivo samples acquired reflecting the morphogen gradients. **a** The *Drosophila* wing imaginal disc diagram. The expression patterns of Ci and Hh specify the A (red region) and P (blue region) compartment of wing imaginal disc, respectively. **b** The diagram of the adult wing. The whole adult wing is derived from the wing pouch region (red and blue colored region) in wing imaginal disc. There are five veins in the wing. Among them, the first three veins (L1–L3) are developed from the A compartment cells and the other two (L4 and L5) are from the P compartment cells. **c** Diagram of the wing imaginal disc section view in A compartment. The dot-line circled region is the area subjected to laser capture. **d**–**d**‴ Immunostaining of a *ptcGal4-*uas*-*GFP wing imaginal disc. GFP signals (**d** and green in **d**‴), Ptc signals (**d**′ and red in **d**‴) and Ci signals (**d**′ and blue in **d**‴). Scale bar, 50 μm. **e**–**k**′ The wing disc sections were cut along A–P axis (bright filed). The circled areas were obtained by laser capture. The wing disc sections before laser capture (**e**–**k**). The wing disc sections after laser capture **(e**′–**k**′**)**. **l**–**p** Real-time qPCR results showing the expression levels of the indicated genes at different positions along A–P axis in a wing imaginal disc. **q** Workflow of Geo-RNA-seq to acquire PGE profiles along A–P axis and bioinformatics analysis.

The *Drosophila* wing imaginal disc is one of the most typical models to investigate the morphogen-mediated tissue patterning in development^4,7,12–14^. In the wing imaginal disc, there exist two axes, the A–P and D–V axes (Fig. 1a)^7,11,12,15^. The tissue patterning of wing disc in both axes is precisely guided by different morphogen gradients and is critical for the wing growth and proper morphology^2,16,17^ (Fig. 1a, c). Generally, tissue patterning in the D–V direction is determined by the Wingless signaling whereas the Hedgehog (Hh) and Decapentaplegic (Dpp) gradients play important roles in tissue patterning along the A–P direction^9,13,18,19^.

In wing discs, Hh is exclusively produced and post-translationally modified by adding the cholesterol and palmitic acid in P compartment cells^7,11,12^, indicating the help of lipoprotein or extracellular structures in Hh transport^8^. Previous reports have shown that Hh patterns the central part of the wing^7,8^, and acts as a morphogen to A compartment cells^11^. When Hh diffuses into A compartment, it binds to Patched (Ptc), the receptor of Hh, and alleviates the inhibition of Smoothened (Smo), and then Ci is changed into active form, which could activate a series of downstream target genes, including *dpp* and *ptc*. Therefore, there exists a secondary effect of Hh gradient in wing disc patterning, which is achieved by activating the expression of Dpp in cells in A compartment near the A/P boundary^11^. In addition, the graded upregulation of Ptc could further restrict the spread of Hh via endocytosis^1^. Moreover, neither the dysfunction nor overexpression of Hh could result in normal adult wing, indicating that Hh gradient is critical for the proper wing disc patterning in *Drosophila*^7,20^.

In addition, organisms are always facing various genetic and/or nongenetic perturbations/alterations during the development and long-time evolution, which have the potential to cause abnormality of tissue patterning and even organism lethality^21,22^. To avoid those detrimental changes, organisms usually possess genetic robustness to tolerate those variations to maintain the phenotypic and functional stability^21,23^. Although it is well known that those morphogen-mediated differential position-related gene expression (PGE) profiles form a tissue-specific gene regulatory network to determine proper tissue pattern, whether morphogen-directed gene expression network possesses genetic robustness remains an open question without systematic investigations.

In the current study, we used the *Drosophila* wing disc as a model system, in which cells established their spatially specific gene expression profiles under the direction of morphogen gradients. We adopted the geographical position sequencing method (Geo-seq) to analyze the regulatory architectures in different positions along the A–P axis. By exploiting the modular and binary features of the measured RNA-seq data, we constructed a morphogen-directed gene modular network (GMN) based on the Boolean model, which is an effective approach for inferring and analyzing biological networks^24,25^. Through in silico and in vivo experiments, we demonstrated that the morphogen-directed GMN is robust in response to various genetic perturbations. Moreover, by combining computational analyses and experimental validations, we demonstrated that this morphogen-directed GMN can also tolerate Hh production changes. Interestingly, Hh production increase seems more likely to be tolerated by this GMN compared to Hh production decrease.

## Results

### In vivo samples acquired by Geo-seq fit the morphogen gradients

To simplify our investigation on the morphogen-mediated genetic robustness, we chose to analyze the PGE profiles exclusively along A–P direction. For this purpose, we developed a GFP reporter fly (*ptcGal4*-uas-GFP) in which GFP expression is driven by *patched* (*ptc*) promoter that responds to Hh gradient. As a result, the cells in A/P boundary is marked with GFP (Fig. 1d–d‴). We then collected the PGE information by adopting Geo-seq approach^26^, a method combining laser capture microdissection^27,28^ with tiny-sample RNA-seq, and performed a series of bioinformatic analyses (Fig. 1q). To precisely collect tissue samples along A–P direction, the GFP-labeled wing imaginal discs were first consecutively cut into 4 μm-thick sections via frozen section along the A–P axis (Fig. 1b), and these 4 μm microdissections were then subjected to precise laser capture to collect tiny tissue masses from the defined section areas (Fig. 1e–k′). This sample collection procedure greatly ensures the PGE profiles derived from those samples faithfully reflecting morphogen gradients along the A–P axis but not the Wingless gradient along the D–V axis. Following this sample collection procedure, we collected a set of frozen sections in the A/P boundary region in the direction of A to P compartment and conducted the laser microdissection to acquire the defined areas (Fig. 1e–k′). The samples are then named with B1–B7 sequentially according to the position of frozen sections from A to P compartment (sample set B). The following qPCR assays demonstrated that *hh* was exclusively expressed in position B7 and *ci* expression was restricted in the region from positions B1 to B6, indicating that position B7 is in the P compartment while the region from B1 to B6 is in the A compartment (Fig. 1l, m). Consistent with previous studies^7,15^, both *ptc* and *dpp* showed morphogen-mediated dynamic level changes. The expression of *ptc* peaked at the A/P boundary (B6) and gradually declined at the positions of A compartment remote from the A/P boundary, and *dpp* was exclusively expressed in the A compartment and reached its expression peak at position B4 (Fig. 1n, o). More importantly, the expression pattern of *gfp* was largely matched with that of *ptc*, which was highly enriched at the A/P boundary region (Fig. 1p). Therefore, these results indicate that the sample collection procedure is proper to investigate PGE profile changes in response to the morphogen gradients along A–P axis.

**Fig. 2:**
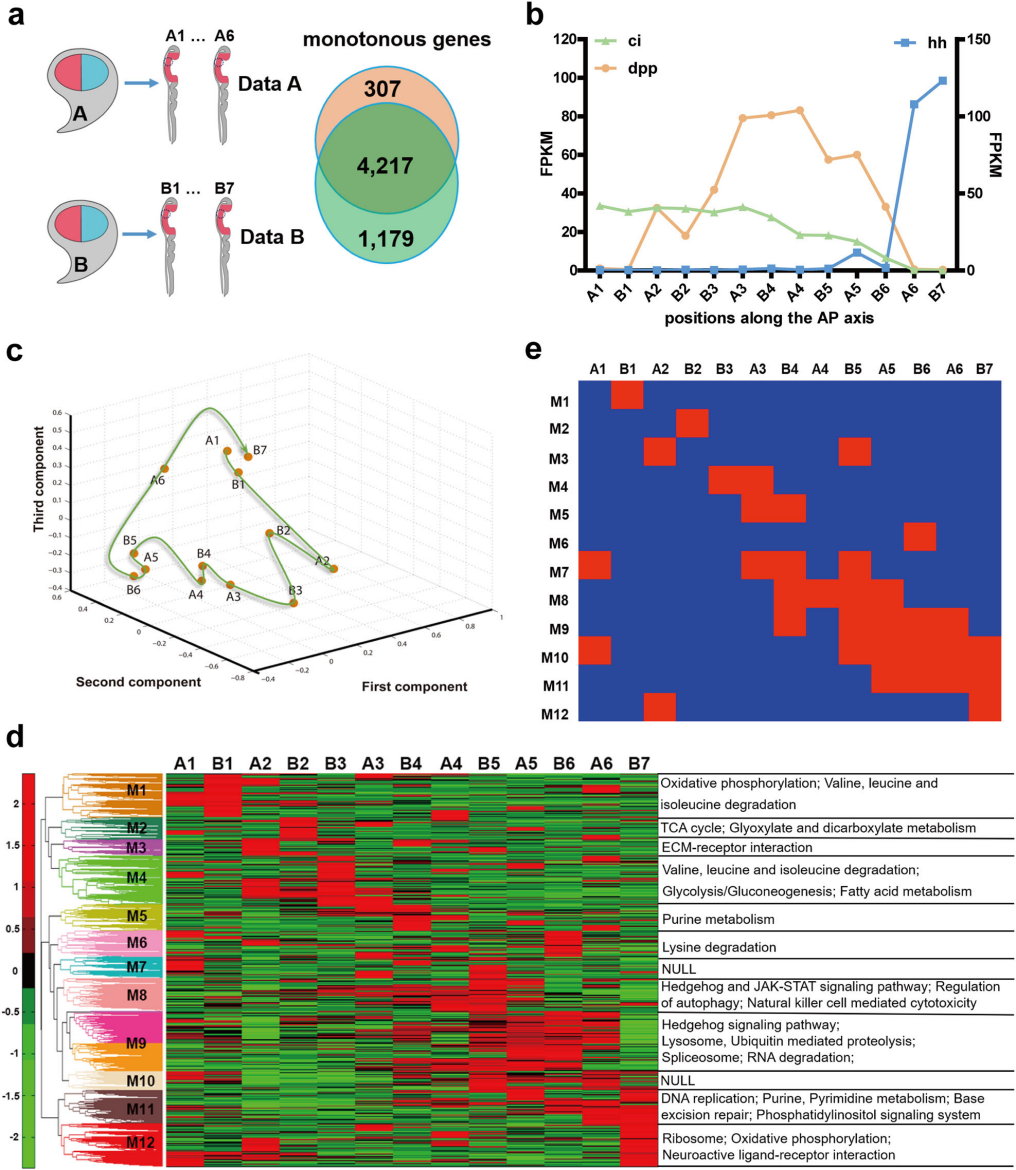
Sample-set integration and binary spatial module state construction. **a** A brief diagram of the sample data collection. Data A is composed of gene information of different positions in wing disc A. There are 4524 MRGs in Data A. Data B is composed of gene information of different positions in wing disc B. There are 5396 MRGs in Data B. There are 4217 common genes in both data sets. **b** Expression dynamics of *hh*, *ci* and *dpp* in sample set integrated with sample sets A and B from RNA-seq data. The Fragments Per Kilobase Million (FPKM) value on the left *Y*-axis represents the expression of *ci* and *dpp*, and the FPKM value of the right *Y*-axis represents the expression of *hh*. **c** PCA of the hierarchical clustering results. The arrow indicates the direction of positions from A to P compartment (A1–B7). **d** Heat map of the expression of 4217 MRGs in integrated sample set. Heat map shows clear position-based modularization of the active genes. **e** Binary module states according to the heat map (Blue indicates inactive and red indicates active).

Following this sample collection procedure, we then collected another set of wing imaginal disc samples from position A1 to A6 in the direction of A to P compartment (sample set A). Because of the diffusible property of morphogen, we hypothesized that genes affected directly by morphogen gradient would show a monotonous trend. Thus, we screened out 4524 genes from sample set A and 5396 genes from sample set B whose expression showed a monotonous trend at least in four consecutive intervals (positions) and defined those genes as putative morphogen-responsive genes (MRGs) (Fig. 2a). After comparison of them, we screened out 4217 common MRGs from both sample sets, which comprise 93.21% and 78.15% of MRGs in sample sets A and B, respectively, indicating the reliability of our RNA-seq data (Fig. 2a). Because the frozen section step of the sample collection procedure carries subjective judgment of operators, it is impossible to collect two sets of samples from exactly the same positions. Instead, each set of samples should be in a staggered position pattern. This staggered pattern between different sample sets actually provided us a good rationale to integrate them into a single sample set, which will carry more refined PGE profile information. Before integrating two sample sets A and B, we first removed batch effect between two datasets by using R package “limma”^29^. Then according to the expression of *hh*, *ci*, and *dpp*, we integrated the A and B sample sets and ordered them as A1, B1, A2, B2, B3, A3, B4, A4, B5, A5, B6, A6, and B7 in the direction of A to P compartment along the A–P axis (Fig. 2b).

### Gene modules derived from MRGs showed a binary expression mode

The position order (A1, B1…to B7) in this integrated sample set was further confirmed by principal component analysis of the 4217 common MRGs (Fig. 2c). To explore the spatial expression patterns of those 4217 common MRGs, we performed hierarchical clustering analysis and found that the activation of them was in a position-dependent manner, i.e., they showed differential responses to the morphogen gradients along A–P axis (Fig. 2d). According to the differential PGE patterns of those 4217 MRGs, we grouped them into 12 modules (M1–M12) (Fig. 2d). Kyoto Encyclopedia of Genes and Genomes (KEGG) enrichment analysis by DAVID^30,31^ of the 12 modules showed that genes involved in the Hh signaling pathway were enriched in the M8 and M9 (Fig. 2d). More importantly, the activation of M8 and M9 was detected at positions B5 and A5, a region close to the A/P boundary, further suggesting the reliability of our RNA-seq data (Fig. 2d). Here we also found that M1, M4 and M6 are mostly enriched with genes involved in the amino acid metabolism, M2 and M4 are enriched with genes linked to glycometabolism, while M11 and M12 are enriched with genes related to purine metabolism, pyrimidine metabolism, and post-translational modification, which are all essential for the basic life activities. However, the genes in M7 and M10 are not specified to given pathways (Fig. 2d).

Two models, binary and graded (or continuous), can be adopted for studying the mechanism of eukaryotic gene induction^32,33^. However, in contrast to the graded induction or continuous dynamics of each individual gene among 4217 MRGs, the activation of all the 12 modules was found to be well presented in a binary manner along the A–P axis based on the RNA-seq data (Fig. 2d, e). The binary pattern of modules implied the effectiveness of the Boolean model in studying this process. Boolean network is one of the simple but effective approaches for constructing various networks and recently has been successfully applied to infer with gene regulatory networks and study the behavior of such binary dynamic networks^24,34,35^. Here, to explore the binary dynamics of modules, we used the Boolean model to study the wing disc development by constructing the GMN.

Boolean network consists of Boolean variables and Boolean functions. For a GMN, each module represents a variable and its value is 1 or 0, which means that the module is active or inactive. The Boolean function indicates the regulatory relations between variables (or modules). To construct the GMN, we first discretized the gene expression data into binary states for each module. We assumed that *s*_i_(*j*) is the state of module *i* in position *j*, where *s*_i_(*j*) = 0 (if module *i* is inactive in position *j*) or 1 (if module i is active in position j). In each module, if the expression value of gene k in position j (denoted as g_kj_) is greater than the average expression of gene k across positions, then we set g_kj_ = 1, otherwise g_kj_ = 0. We set *s*_i_(*j*) = 1 if more than half of the genes in position *j* are 1 for module *i*, otherwise *s*_i_(*j*) = 0. Then we obtained a vector *S*(*j*) = (*s*_1_(*j*),*s*_2_(*j*),...,*s*_12_(*j*)), which is the spatial state of all 12 modules for position *j* in the system, where the position *j* = 0, 1, 2, …, *N* with *N* + 1 = 13 positions. Hence, *S* = (*S*(0),*S*(1),...,*S*(*N*)) represents the states for all the modules across different positions (Fig. 2e and Supplementary Table S1). However, given the fact that the concentration of the major A–P axis morphogen, Hh, is almost the same in the P compartment^7,12^, we assumed that the module pattern at the position right next to the B7 would be constant. Hence, we added B7*, a duplication of B7, on the spatial diffusion data as the final state, and analyzed the dynamic process of the binary module map by using S^*^ = (*S*(0),*S*(1),...,*S*(*N*),*S*(*N* + 1)) to represent the binary spatial module states along the A–P direction (Supplementary Table S2).

### The GMN constructed by Boolean model reflects the dynamic interactions between modules

To analyze whether morphogen-directed development possesses genetic robustness, we first constructed a GMN based on this established binary module states along the A–P axis by Boolean model^36–38^ (Supplementary Table S2). For this purpose, we set the concentrations along Hh gradient as time variables in Boolean model. Thus the spatial development patterns of wing disc can be considered as a time-based dynamic process of modules. In this respect, each position *j* represents a time point, while a module pattern *S*(*j*) is the state of that time point. Specifically, we used the direction from A to P compartment as the direction of time, and regarded the positions from A1 to B7* as time *j* from 0 to 12 + 1 due to the additional B7*^39^. Then we used the binary module spatial data S^*^ (Supplementary Table S2) to construct a GMN based on Boolean model (i.e., *f*_i_ in Eq. (1)) to reveal the dynamic process of the transcriptome along the A–P axis as follows^24,34,40–42^. There are 12 modules (M1,…,M12), and for module i, we have the following modular dynamics

1 $$s_{\mathrm{i}}(j + 1) = f_{\mathrm{i}}(s_1(j),s_2(j), \cdots ,s_{\mathrm{m}}(j)),$$

where module *i* = 1,…,*m* (*m* = 12 is the number of the modules), and position *j* = 0,…,*N* + 1 (*N* + 1 = 13), and *f*_i_ is the Boolean function or modular interaction to module *i* from other modules, which is derived from the observed dataset. Specifically, *f*_i_ is composed by three operations^39,43^, AND, OR, NOT, which determine the regulation rules of other modules to module *i*, such as *f*_1_ for M1 is (NOT M9) AND M10 AND (NOT M12), or $f_1 = \overline {M9} \cap M10 \cap \overline {M12}$. Based on the Eq. (1) and the observed data, we can get the solution or regulation rule for each *f*_i_ by using R package BoolNet^36^, and obtain the whole regulation rules, which are given in Supplementary Table S3. Then, we can construct the GMN based on Supplementary Table S3.

**Fig. 3:**
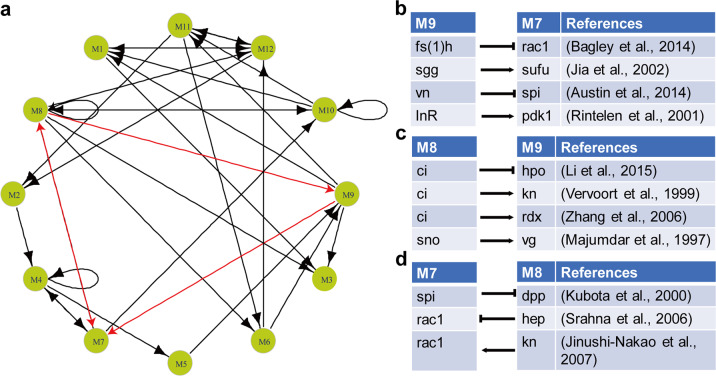
GMN constructed by Boolean model. **a** The GMN based on Boolean model. M3 is the regulatory terminus of the network, which is directly regulated by the M1, M8, and M9. M7, M8, and M9 form a regulatory triangle at the center of the GMN (the triangle with red outline). **b**–**d** The identified gene regulatory interactions among M7, M8, and M9 from previous reports.

In this GMN, M3 is the regulatory terminus of the network (Fig. 3a). Interestingly, M3 has been classified as a module with enriched genes related to extracellular matrix–receptor interaction (Fig. 2d), which have been reported to make important contributions to wing disc development^44^. More importantly, systematical analysis showed that three modules, M7, M8, and M9, form a regulatory triangle that is at the central position of this GMN (Fig. 3a), and both M8 and M9 are hubs with more than six links in the network, further indicting the importance of these modules in the network (Fig. 3a). Among the central regulatory triangle, M7 and M8 can regulate each other directly and M8 can also indirectly regulate the M7 via M9 (Fig. 3a), in which the gene regulatory interactions are supported by previous studies (Fig. 3b–d). These further suggested the reliability of our GMN result. This regulatory triangle shows that there exists internal reciprocal regulation, which may contribute to the stability of the GMN and buffer the fluctuations in the regulatory network. In addition, given that genes involved in the Hh signaling pathway are enriched in both M8 and M9 (Fig. 2d), this GMN also indicates that Hh signaling is under precise regulation during the development of *Drosophila* wing discs. Moreover, the importance of such precise regulation on Hh signaling has been confirmed by our experimental data, in which abnormal adult wings were formed in the circumstances of artificial upregulation, knockdown, expression of active or inactive mutants of *ci* or *smo* (Supplementary Fig. S1).

### There exist three attractors in the GMN

**Fig. 4:**
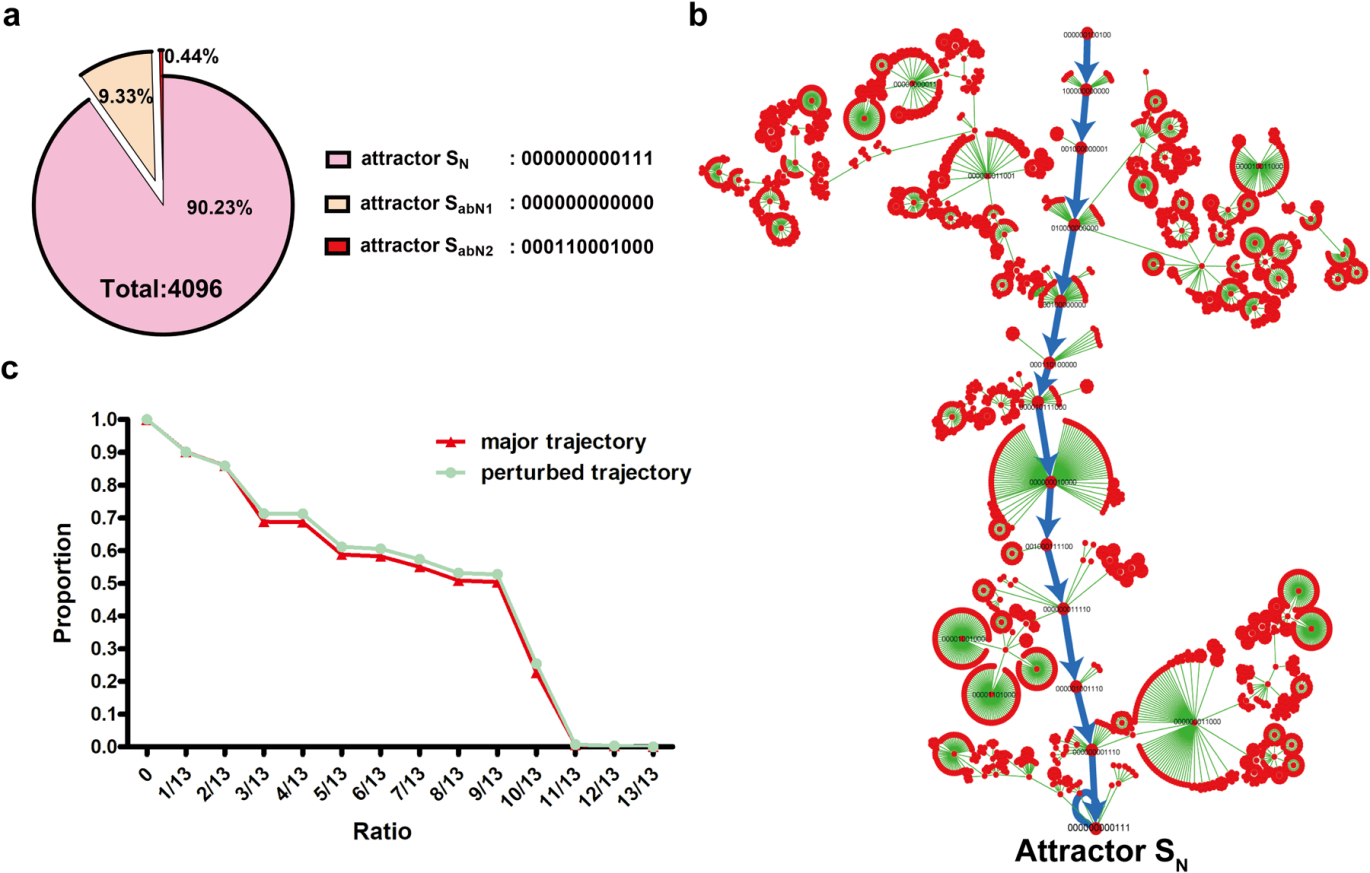
Computational analyses and biological validation of the GMN robustness of *Drosophila* wing imaginal disc. **a** The attractors of GMN. There are three attractors: attractor normal (*S*_N_, 90.23%), attractor abnormal 1 (*S*_abN1_, 9.33%) and attractor abnormal 2 (*S*_abN2_, 0.44%). **b** The biggest state-transition tree converged to the attractor *S*_N_. The major (physiological) trajectory is labeled with blue lines connected with 13 physiological states along A–P axis. **c** The distribution of the state overlap ratios between all the other possible trajectories and the major trajectory. The red line is for the original network. Green line is for the randomly perturbed network.

To further study the behavior and dynamic properties of the GMN, we performed the attractor analysis of this GMN by BoolNet package^45^. The attractor represents a steady state/states of the Boolean network, toward which the network tends to evolve (Supplementary Table S4 for details). The number of states converging to an attractor is called the basin size of this attractor, and the larger basin size indicates the more stable network of this attractor^34^. Here, the basin of an attractor is all of those states which leads to this attractor (Supplementary Table S4). Since we have 12 modules, the GMN has 2^12^ possible states. Attractor analysis showed that total 2^12^ = 4096 possible initial states S(0) converged to three attractors: 3696 (90.23%) in normal (attractor *S*_N_ = 000000000111), 382 (9.33%) in abnormal 1 (attractor *S*_abN1_ = 000000000000) and 18 (0.44%) in abnormal 2 (attractor *S*_abN2_ = 000110001000) (Fig. 4a). More importantly, the module pattern of attractor *S*_N_ is exactly the same with that of the P compartment in wing disc, whereas the patterns of *S*_abN1_ and *S*_abN2_ are not. In addition, there are not significant correlations between those two abnormal attractors with specific module alterations after analysis of the module patterns of all possible initial states *S*(0) which led to *S*_abN1_ and *S*_abN2_ (Supplementary Fig. S2a, b). The above indicates that most (>90%) of the 2^12^ = 4096 possible initial states *S*(0) converge to a single attractor *S*_N_ corresponding to the normal physiological module pattern of *Drosophila* wing disc, suggesting that this GMN is robust.

### The genetic robustness of the GMN

To determine the robustness of this GMN, we analyzed the dynamic properties of the GMN via the state-transition tree for all possible initial states (2^12^ = 4096)^34,41,46^. Here, the state-transition tree of an attractor is all of those trajectories which lead to this attractor (Supplementary Table S4), and each node in the tree indicates a state of GMN, while the directed lines represent transitions between two states. We acquired three state-transition trees along A–P direction, corresponding to three attractors, respectively. Among them, the biggest one converged to the attractor *S*_N_ (000000000111) (Fig. 4b), whereas the other two converged to *S*_abN1_ and *S*_abN2_, respectively (Supplementary Fig. S2c, d). Interestingly, the total 13 states on the major trajectory of the biggest tree are concordant with the physiological data, i.e., A1,B1,…,B7 (Supplementary Table S4; Fig. 4b, the red dots connected with blue lines) and we then named the major trajectory (or major path) as physiological trajectory hereafter. Note that a trajectory of the GMN corresponds to a wing disc developmental path, which is also a path of states from an initial state to an attractor.

To further strength the evidence for the robustness of this GMN, we made random perturbations on state-transition table of the GMN in the original spatial diffusion table (Supplementary Table S2). We randomly flipped two bits in the original diffusion table (Supplementary Table S2) and calculated the attractor and the transition tree for the perturbed GMN, we did this perturbation 1000 times. These perturbations will not alter the structure of the Boolean network but may change the trajectory^47^. We found that 99.92% of the 1000 perturbed GMN have the same attractors as the original GMN, which were 000000000111 (3658 states on average, 89.31%), 000000000000 (420 states on average, 10.25%) and 000110001000 (18 states on average, 0.44%) (Supplementary Fig. S3a). More importantly, similar to the original GMN, almost 90% of the possible initial states S(0) in the perturbed GMN are on a state-transition tree, including the major trajectory, which is concordant with the physiological trajectory. This result means that the perturbation does not alter the original major trajectory (Fig. 4b and Supplementary Fig. S3b). This result also demonstrated that the GMN is stable or dynamically robust to various alterations^47^. To further illustrate robustness of the trajectory, we calculated and compared the distribution of the states converging to the attractor *S*_N_. Computational analysis of the distribution of states between the physiological trajectory and other possible trajectories (i.e., the trajectory from other possible initial state to attractor *S*_N_) displayed nearly the same distribution patterns between the original GMN and the perturbed one with *P*-value of 1.9903e−18 (Fig. 4c, red line vs. green line; detailed method can be found in Supplementary methods). In other words, these findings collectively indicate that the pattern formation governed by morphogen-directed GMN in *Drosophila* wing discs is significantly robust because the random perturbations in the GMN may frequently lead to organ patterns similar to the physiological one.

To validate the robustness of the GMN experimentally, we performed state perturbation (biological) tests by randomly changing the expression of 30 genes (genes were selected from Supplementary Table S5). Those alterations would change the physiological initial state (A1) (000000100100) to 9 possible initial states *S*(0) (the module patterns of the nine possible initial states *S*(0) were presented in Supplementary Fig. S4). Compared with the physiological initial state (A1) (000000100100), each of those nine possible initial states *S*(0) carries only one module state difference (ON or OFF) (Supplementary Fig. S4). By employing the *tubGal80*^*ts*^; *ciGal4* to specifically turn on (overexpression) or off (RNAi knockdown) those genes in A compartment in the third instar larvae stages, we introduced the nine possible initial states *S*(0) into the wing disc and examined whether those alterations could change the *Drosophila* wing pattern. Before we detected the adult wing phenotype, RNAi or overexpression efficiency in the third instar larvae stage was confirmed (Supplementary Figs. S5–S8). We then detected the adult wing phenotype, and found that most perturbations would not change the vein pattern and the morphology of the adult wing. Finally our biological validation demonstrated that only 3 out of 30 (10%) gene expression alterations resulted in abnormal adult wing phenotypes (Supplementary Figs. S9 and S10), indicating that the morphogen-directed GMN is robust in response to genetic perturbations.

**Fig. 5:**
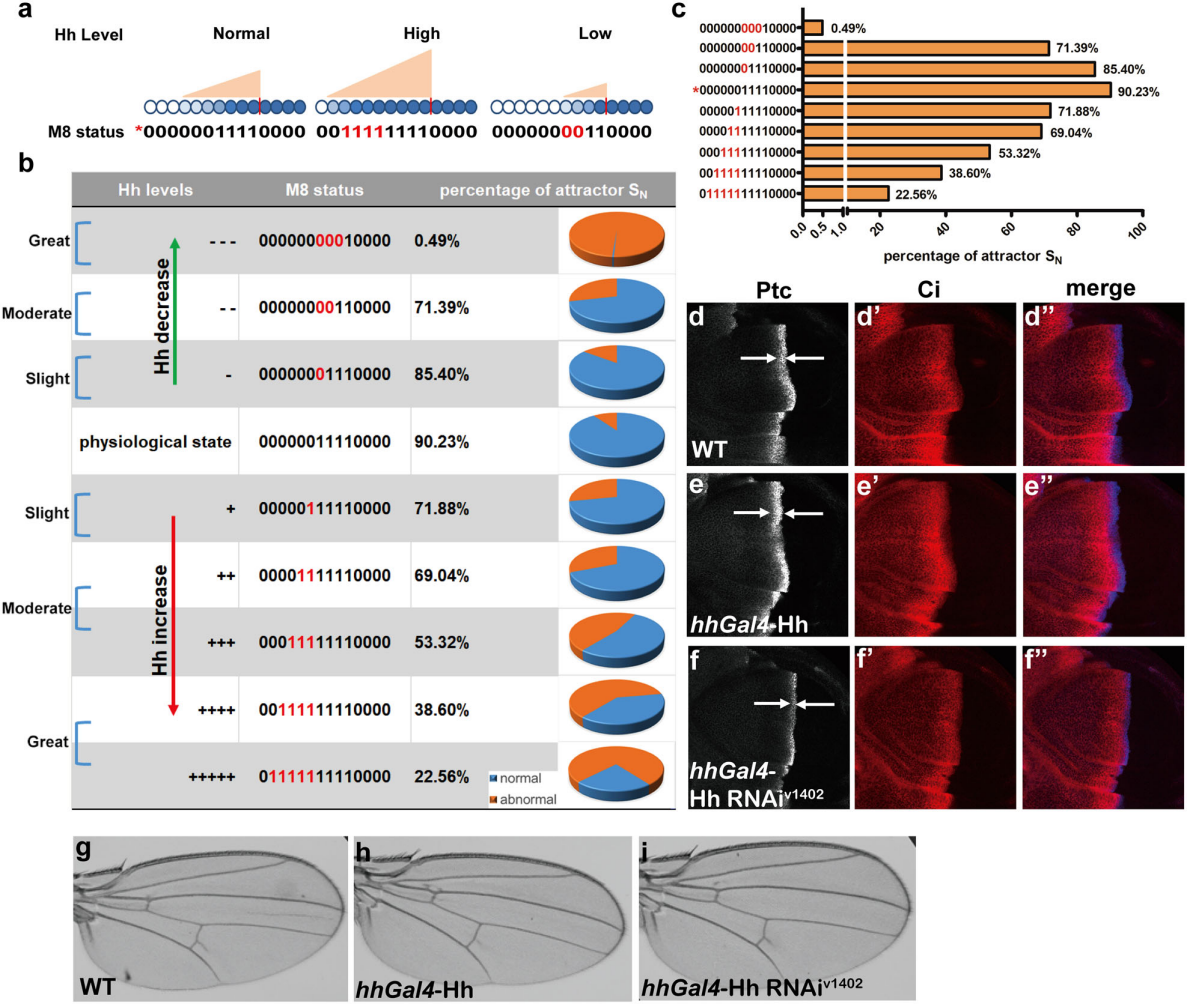
Attractor analysis in response to Hh production changes. **a** Hypothesis model of how Hh production changes affect the induction status of M8 along A–P axis. **b** In response to Hh production changes, the ON or OFF status of M8 at different positions is changed accordingly. Percentage of the normal attractor (attractor *S*_N_) was calculated based on the corresponding M8 changes. **c** The histogram of the percentage of normal attractor *S*_N_ in response to Hh production changes. The vertical coordinate indicates the variations of M8 in response to different Hh levels. The asterisk indicates the physiological state of M8. The upper states of M8 representing the Hh level decrease. The lower states of M8 representing the Hh level increase. The *X* axis showed the percentage of *S*_N_. **d**–**f″** Wing discs with the indicated genotypes were immunostained with anti-Ptc (blue) antibody or anti-Ci (red) antibody. Ptc and Ci signals were presented in *Drosophila* wing discs with WT (**d**–**d″**), Hh overexpression (*hhGal4*-uas-Hh) (**e**–**e″**), or Hh knockdown (*hhGal4*-Hh RNAi^V1402^) (**f**–**f″**) genotypes. The width changes of Ptc-positive regions (between two white arrows) indicate the corresponding Hh gradient alterations (**d**–**f**), which is also in accordance with the Ci change (**d**′**, e**′ and **f**′). **g**–**i** Morphology of *Drosophila* adult wings with the indicated genotypes. An adult wing of WT control (**g**), an adult wing of *Drosophila* with Hh overexpression that is driven by *hhGal4* in third instar larvae stage (**h**), an adult wing of *Drosophila* with reduced Hh production that is achieved by *hhGal4*-Hh RNAi^V1402^ in third instar larvae stage (**i**).

Since we have tested the GMN robustness in the condition of genetic alterations (Supplementary Figs. S9 and S10), we then sought to analyze the GMN robustness in response to nongenetic alterations, such as the Hh level changes. Here, we used the M8 as a readout of the levels of Hh production because genes related to the Hh signaling pathway are enriched in this module (Fig. 2d). When the P compartment cells produce more Hh ligands, the range of Hh gradient in the A compartment of wing disc should be much wider than the physiological one, which will lead to M8 induction at the positions far away from the A/P boundary (Fig. 5a). By contrast, when the Hh production is low in the P compartment, the range of Hh gradient is supposed to be narrow, which will subsequently turn off M8 at the positions close to the A/P boundary (Fig. 5a, detailed in Supplementary methods). By computing the percentage of possible initial states *S*(0) that converged to the normal attractor (attractor *S*_N_), we found that more than 50% of possible initial states *S*(0) converged to attractor *S*_N_ in the condition of slight or moderate Hh production changes (Fig. 5b, c). In this analysis, we also found that this GMN is friendly to Hh production increase rather than production decrease. As shown in Fig. 5b, c, Hh production increase led to a gradual decline in the percentage of possible initial states *S*(0) which could converge to attractor *S*_N_, whereas severe Hh production decrease somehow completely abolished the convergence of possible initial states *S*(0) to attractor *S*_N_. Therefore, these findings indicate that the GMN is also robust in response to nongenetic perturbations such as Hh production alterations.

To experimentally confirm the GMN robustness in Hh production alterations, we adopted the *hhGal4* to drive Hh overexpression or knockdown to respectively increase or decrease the Hh production in P compartment in third instar larvae stage. We first detected the Ptc and Ci levels in different Hh conditions (Fig. 5d–f″). The Hh production increase widened the Ptc-positive region along the A–P axis, whereas the Hh production decrease narrowed it compared with the wild-type (WT) control (Fig. 5d–f″), indicating the stretching or shrinkage of the Hh gradient. Correspondingly, Ci levels were increased or decreased in response to Hh gradient changes (Fig. 5d′–f′). As we expected, the adult wing showed no obvious morphology changes despite the apparent Hh gradient alterations, which further underlines the robustness of the morphogen-directed GMN in response to nongenetic alterations (Fig. 5g–i).

## Discussion

Precise tissue patterning is essential for normal biological function and phenotypic stability in multicellular organisms, and the morphogen gradient plays an indispensable role in the proper tissue patterning. Although the mechanisms of morphogen-directed signal transduction have been extensively interpreted, the mechanism of how morphogen achieves constant and precise tissue patterning remains unclear. A recent study indicates that the double-negative regulatory logic and the negative feedback of Hh signaling receptor Ptch1 are important for Hh gradient formation and robustness to variations of morphogen production^48^, suggesting that morphogen-directed precise tissue patterning can be established through regulating extracellular morphogen signal inputs. Instead of those extracellular regulatory mechanisms to ensure cells to receive stable and constant morphogen signals in the situation of morphogen production changes, there is a long-standing but yet not fully validated “common sense” believing that morphogen-directed intracellular genetic network may possess robustness to guarantee proper tissue patterning in response to various perturbations including morphogen production variation and genetic alterations. Here, we combined Geo-seq, mathematical model and biological validation to investigate whether morphogen-directed genetic network possesses robustness in *Drosophila* wing disc. We demonstrated that the morphogen gradient-directed genetic network along A–P axis of wing disc is robust in response to either genetic or nongenetic alterations.

In this study, we found that the activation of gene modules that contain clusters of the MRGs showed a binary dynamics from A to P compartment via Geo-seq. Thus, we used Boolean model to construct the whole GMN by exploiting this binary modular pattern according to the data heatmap. The advantage of this GMN is able to identify the core regulators among thousands of genes because Boolean model can act as Occam’s Razor to simplify the network structure by exploiting such binary modular dynamics, but we caution that this method is still subject to a potential pitfall that the Boolean model-based modules may inevitably lose the details of the gene–gene interaction.

Moreover, the GMN based on the Boolean model showed that there is a central regulatory triangle, which is composed of M7, M8 and M9 (Fig. 3a). KEGG pathway enrichment analysis further showed that several pathways important for wing disc patterning such as Hh signaling, JAK-STAT pathway, ubiquitination and autophagy, are enriched in this regulatory triangle. Furthermore, the activation of these three modules is mainly located near the A/P boundary (Fig. 2d, e, positions from B5 to A6), indicating that the activation of these signal transductions is critical for the wing disc development. In addition, the mutual regulations in the regulatory triangle showed that there are internal self-regulations to keep the proper tissue pattern in the development, which may be the source of the robustness in *Drosophila* wing disc.

We then performed the attractor analysis to investigate the dynamical properties (or asymptotical properties) of this GMN. We first analyzed the attractor in the direction from compartment P to A in line with the diffusion direction of the Hh ligand. Consequently, we computed a sole attractor, 000000000000, which means that all modules are silenced in the A compartment. This module pattern is apparently not consistent with the biological status in the A compartment. Then, we computed the GMN attractor at the direction from A to P compartment. There are three attractors including *S*_N_, *S*_abN1_, and *S*_abN2_ generated from this GMN. The module pattern of attractor *S*_N_ is exactly the same as its physiological counterpart of the P compartment whereas the patterns of *S*_abN1_ and *S*_abN2_ are not. In other words, through this GMN, most (>90%) of the 4096 initial states *S*(0) converge to a single attractor *S*_N_ corresponding to the normal physiological pattern of *Drosophila* wing disc, indicating the robustness of the GMN. Here, we have to admit that the attractor analyses in either A-to-P or P-to-A directions may not fully and physiologically reflect the biological process of wing disc development because the gene–gene regulatory interaction in response to the Hh gradient during wing disc development is still an open question.

Moreover, we further analyzed the dynamic properties of the constructed GMN via the state-transition trajectory for all possible states. The biggest trajectory converged to the attractor *S*_N_ (000000000111), which is consistent with the original biological status and its basin covers over 90% of states, indicating the robustness of the GMN. Furthermore, we also made random perturbations on states in the original GMN to test the GMN robustness. Each perturbed GMN has the same attractors, major trajectory and similar distribution of the state overlap ratios to the original physiological GMN (Fig. 4c and Supplementary Fig. S3). Moreover, by randomly changing the gene expression levels in nine possible initial states *S*(0) of this GMN, we biologically confirmed that the GMN well tolerates most genetic alterations (Supplementary Figs. S4, S9, and S10). However, each module contains hundreds of genes, it is impossible to change the status of all genes simultaneously, we also realized that the single gene alteration including mutation, overexpression or downregulation is almost impossible to change the on or off status of the indicated modules no matter how closely connected with other genes or how important it is. Nevertheless, we do see the development of *Drosophila* wing can tolerate most of gene alterations suggesting the robustness of gene network during *Drosophila* development. In addition, by computing attractors in the condition of artificial Hh production changes, we demonstrated that slight or moderate Hh production changes (either decrease or increase) can be well tolerated by this GMN (Fig. 5). Therefore, by combining computational analyses and biological experiments, we for the first time demonstrated that the morphogen-directed GMN in *Drosophila* wing disc is robust in response to both genetic and nongenetic alterations.

In summary, we adopted the *Drosophila* wing imaginal disc as a model and combined Geo-seq method with bioinformatic analysis to systematically analyze the dynamics of morphogen-directed gene regulatory networks. Here, according to the spatially specific MRG expression profiles of wing discs, we established a morphogen-directed GMN. By both computational analyses and biological validation, we further demonstrated that the GMN in *Drosophila* wing disc is robust in response to various genetic perturbations, which ensures the phenotypic stability of their wing discs and wings. Moreover, by computationally mimicking the Hh production changes, we demonstrated that this GMN can well tolerate a certain range of Hh production changes. Interestingly, this GMN seems more likely to tolerate Hh production increase rather than production decrease (Fig. 5), suggesting that deficiency of Hh production is more detrimental for wing disc development in *Drosophila*. In addition, the Mathematics-Biology integrative approach employed in the current study can also be adopted to analyze the genetic robustness of other types of tissues in diverse organisms.

## Materials and methods

### Wing disc sample preparation

We adopted the *ptcGal4-*uas-GFP transgenic fly to indicate the A/P boundary in wing discs from the third instar larvae^13^. All flies were raised in the standard medium at 25 °C unless otherwise indicated. All tools and reagents were RNase free. The third instar larvae were cut in half and wing discs were dissected and fixed in ethanol for 45 s, then imbedded into the OCT (Lecia) on the slide at −80 °C overnight. On the second day, the wing disc samples were imbedded again into the mold and frozen overnight. After that we conducted the cryosection (Lecia CM 3050s) and cut consecutive 4-μm-thick sections along the A–P axis with the GFP fluorescence guidance. Then, the disc pieces were fixed on the PEN membrane slide (Cat# 50102, MMI), treated by 70% ethanol for 90 s, and then 100% ethanol for 90 s. After these, the wing disc pieces far away from the DV axis were cut by the CellCut laser microdissection system (CellCut System, MMI), by which we could get the correct samples in the defined areas without the Wingless effect.

The samples obtained via the laser capture were then prepared for the RNA extraction by dissolving in 50 μL of 4 M GuSCN (Cat# 15577018, Invitrogen), incubated at 42 °C for 15 min and centrifuged for 3 min at 4 °C. The supernatant was transferred into a new 1.5 mL tube, and 771 μL precipitating buffer (78% ethanol, 0.04 M sodium acetate, pH 6.5, 20 μg Glycogen) was added and mixed thoroughly. The samples were incubated at −80 °C overnight. Then the samples were centrifuged, and the pellet was washed by 70% ethanol once and redissolved in solution buffer (1 μM 3′ CDS primer^26^, 1 mM dNTP and 2U RNase inhibitor dissolved in the nuclease-free water). Purified RNA samples were then used for reverse transcription using the SuperScript II reverse transcriptase (Cat# 18064-014, Invitrogen). Acquired cDNA was further amplified using the KAPA HiFi HotStart ReadyMix (Cat# KK2601, KAPA Biosystems). The amplified cDNAs were then sent for sequencing by Bohao Bio Company after the qPCR quality evaluation^26^.

For the quality evaluation, 2 μL cDNA from each sample were diluted by 20-fold with nuclease-free water and further used for qPCR (1 μL diluted cDNA per 10 μL reaction mix) to check the quality using the SYBR Green Mix (Cat# QPK-201, Toyobo) on BioRad CFX96 system. 2^−ΔΔCt^ method was used for the relative quantification with *rpl32* as internal control. Data were presented as means ± SEM, *n* ≥ 3. The primers used for qPCR were as follows (5′ to 3′):

*rpl32*: CTAAGCTGTCGCACAAATGG; AGGAACTTCTTGAATCCGGTG

*dpp*: GGCTTCTACTCCTCGCAGTG; TAATGCTGTGCTGGTCGAGG

*hh*: CTCTTCATGGACCGCAACCT; AACGTGAGCTTCTGGCTCTC

*ci*: CAAATGCACGTTTGAAGGCTG; ATCCCGGATACTCGCAAGTG

*ptc*: TGGACAAGGAACTGGTGCTC; CAATTTGCCCTGAGAAGCTCC

*gfp*: ACGTAAACGGCCACAAGTTC; AAGTCGTGCTGCTTCATGTG

### Clustering gene expression data and constructing gene modules

We generated two datasets of gene expression, Data A and Data B, from two independent biological experiments, and both datasets contain 15,016 genes. In dataset A, we detected six positions in the wing disc from A compartment to P in sequence, which were A1, A2, A3, A4, A5, and A6. Similarly, for dataset B, we detected seven positions in order, namely B1, B2, B3, B4, B5, B6, and B7. Firstly, we selected the genes which have the trend of monotonousness in consecutive four positions. Particularly, we selected gene *k* (denoted as *g*_k_) if the expression of *g*_k_ satisfies the following condition in consecutive four positions

$$g_{\mathrm{k}}(j) \ge 2g_{\mathrm{k}}(j + 1),\,\,{\mathrm{or}}\,g_{\mathrm{k}}(j) \le 2g_{\mathrm{k}}(j + 1),$$

where *j* represents position, *j* + 1 represents the position next to *j* from A to P compartment. Through the above criterion, we screened out 4524 genes in dataset A and 5396 genes in dataset B. Finally, we selected 4217 common genes in both datasets. Thus, we used these 4217 genes for further analysis.

Before further study, we removed the batch effect of two datasets using function removeBatchEffect in R package “limma”^29^ and then integrated two datasets with the expression of *hh*, *ci*, and *dpp* as described in the main text. Thus, we obtained a combined dataset with 4217 genes and 13 positions where we use *j* = 0,1, …, *N* (here *N* + 1 = 13) to denote the positions A1, B1, A2, B2, B3, A3, B4, A4, B5, A5, B6, A6, and B7 with a total of 13 positions. We clustered the 4217 genes into 12 gene modules, named M1, M2,…, M12, according to the hierarchical clustering result. In each module, if the expression value of gene *k* in position *j* (denoted as *g*_kj_) is greater than the average expression of gene *k* across positions, then we set *g*_kj_ = 1, otherwise *g*_kj_ = 0. Then the state of each module can be estimated in a binary manner. Specifically, assume that *s*_i_ (*j*) = 0 or 1, where *i* = 1, 2, …, *m* (here *m* = 12 modules) and *j* = 0,1, …, *N* (here *N* + 1 = 13 positions), is the state of module *i* in position *j*. We set *s*_i_(*j*) = 1 if more than half of the genes in position *j* are 1 for module *i*, otherwise *s*_i_(*j*) = 0. Then a vector *S*(*j*) = (*s*_1_(*j*),*s*_2_(*j*),…,*s*_m_(*j*)), where *j* = 0, 1, 2,…, *N*, is the state of all modules in position *j*. Let *S* = (*S*(0),*S*(1),…,*S*(*N*)) represent the spatial states of all modules for the Hh gradient system and be also the combined dataset of gene modules for further network analysis.

### GMN based on Boolean model

For a set of variables $\{ s_1,s_2, \cdots ,s_{\mathrm{m}}\}$, let each variable *s*_i_ = 0 or 1 be binary, the GMN based on Boolean model is defined as Eq. (1) or as follows:

$$s_{\mathrm{i}}(j + 1) = f_{\mathrm{i}}(s_1(j),s_2(j), \cdots ,s_{\mathrm{m}}(j)),$$

where *j* = 0, 1,…, represents the time points, and *f*_i_ is the Boolean function or modular interaction to module *i* from other gene modules, which can be derived from the observed dataset. The Boolean equation above or Eq. (1) means that the value of gene module *s*_i_ at time *j* + 1 is determined by the other variables at time *j* (including *s*_i_ at time *j*) by means of Boolean function *f*_i_. Here we regarded the position as time point due to the development process of the wing from A to P compartment, and thus we can utilize GMN to analyze the wing growth based on the gene modules and their cross-position regulations. First of all, based on the biological experiments, the Hh concentration will be constant in the P compartment and the states of all the modules on the right of B7 will be the same as in position B7. Hence, we added B7* (*S*(*N* + 1)), a duplication of B7 (*S*(*N*)), on the spatial diffusion or development data as the final state (Supplementary Table S2), and then the dynamic process of modules would be from A1, B1, A2,…, to B7 and B7*, which is thus as the observed sequence or dataset of the gene modules (states) for the network inference of the Hh gradient system. Then we used R package BoolNet to construct the Boolean network or GMN Eq. (1) based on the dataset of the gene modules.

### Attractors and trajectories of GMN

To study the dynamic property of the modular network, we calculated the trajectory of the GMN Eq. (1) from each initial value *S*(0) = (*s*_1_(0),*s*_2_(0),…,*s*_m_(0)), and also its attractor. If there is a time point *T*, and when time *j* is equal or greater than *T*, for all the variables $s_{\mathrm{i}} \in \{ s_1,s_2, \cdots ,s_{\mathrm{m}}\}$, the following is true,

$$s_{\mathrm{i}}(j + 1) = s_{\mathrm{i}}(j),$$

then, $S(T) = (s_1(T),s_2(T), \cdots ,s_{\mathrm{m}}(T))$ is the attractor of the GMN or Boolean network for gene modules. Intuitively, an attractor is a state of modules converged from the initial state. A trajectory of the GMN is a path of states from a specific *S*(0) to an attractor *S*(T) (details in Supplementary Table S4).

We applied Eq. (1) by BoolNet on all possible 2^12^ = 4096 initial states as *S*(0). Finally, all of these 4096 states converged to three stable stationary states, namely, attractor *S*_N_ = 000000000111, attractor *S*_abN1_ = 000000000000 and attractor *S*_abN2_ = 000110001000. The basin sizes for these three attractors are 3696, 382, and 18, respectively. In other words, among all 4096 states, there are 3696 initial states *S*(0), in which all converge to the same attractor *S*_N_, 382 initial states converged to the attractor *S*_abN1_ and 18 initial states converged to *S*_abN2_. Despite different initial values, clearly most (>90%) of the states converge to the attractor *S*_N_ = 000000000111, which is the biggest stable stationary state and also corresponds to the normal state of wing disc. Through applying Eq. (1) on all the possible 4096 states, we can also obtain the state-transition table for three attractors respectively, which are state-transition graphs or trees as shown in Fig. 4 and Supplementary Fig. S2. Each red node represents a state of 12 modules, and the directed line represents the state transition from one state to the next. Clearly, most initial states reach the major trajectory (i.e., the path of the normal wing disc development, as shown in Fig. 4) which also leads to the biggest and main attractor *S*_N_. This implies the robustness of the normal wing disc development even with various perturbations.

### Statistics

All of the statistical analyses are one-tailed, unpaired, *t*-tests with equal variances. All of the experiments are repeated three independent times with similar results unless stated otherwise. Images shown are the representative of images obtained, which are not less than 5.

## Supplementary information

Supplementary Information

Supplementary Table S5

## Data Availability

The list of MRGs in each modules is shown in Supplementary Table S5.

## Supplementary information

**Supplementary Information** accompanies the paper at (10.1038/s41421-020-0173-z).
