# Supplementary material for: Analysis on gene modular network reveals morphogen-directed development robustness in Drosophila
Source: Cell Discov. 2020 Jun 30;6:43. doi: 10.1038/s41421-020-0173-z (PMC7324402; doi:10.1038/s41421-020-0173-z)
Supplement: Supplementary file 1 — Supplementary Information [file 41421_2020_173_MOESM1_ESM.pdf]

Supplementary Materials and Methods

Drosophila strains

Drosophila strains used in this study were maintained under standard conditions unless stated otherwise. The yw flies were used as the controls. The *ptcGal4*, *dppGal4*, *ciGal4*, *hhGal4*, uas-GFP, uas-Hh, uas-Flag-Smo-WT, uas-Flag-SmoSD123, uas-Flag-SmoPKA, uas-Ci103, uas-Ci101, uas-Ci76, uas-Ptc, uas-Eff, uas-PcWT, uas-Ubx, uas-Yki and uas-Fs(1)h have been described before (Flybase)[1-7]. The uas-Antp (BS7301), uas-Hep^{CA} (BS6406), uas-Tkv-GFP (BS51653), uas-Hsp23 (BS30541), S6k RNAi (BS41702), uas-Actin5C (BS24777), uas-Ulp1 (BS44380), uas-αTub84B (BS23861), uas-mir-1012 (BS61495), uas-HT1B (BS27633), uas-Sc (BS26687), uas-Hbs (BS41797), uas-Fwe (BS51611), uas-Drl (BS64333), Sufu RNAi (V35055), Rac1 RNAi (V49247), Spi RNAi (BS28387, BS34645, V3920), Hh RNAi (V1402), Smo RNAi (NIG, 11561R-1), Ci RNAi (NIG, 2125R-1), uas-En (Kyoto 106656) were obtained from Bloomington, VDRC, NIG or Kyoto Stock Center. The uas-Rho1 and uas-E2f1 transgene flies were gifts from Dr. Wei Du. The uas-Cnc transgene fly was a gift from Dr. Haiyun Song.

Immunostaining

For immunostaining of wing imaginal discs, third-instar larvae were cut in half, turned over and fixed in freshly made 4% formaldehyde in PBS at room temperature for 30 mins, and then washed three times with PBST (PBS, 0.1% Triton X-100) for 15 mins. Larvae were then incubated overnight with

primary antibody diluted in PBST at 4 °C, and then washed three times for 20 mins with PBST and incubated with secondary antibody diluted in PBST for 1 hour at room temperature in dark. After washing, wing imaginal discs were dissected and mounted in 40 % glycerol. Leica LAS SP8 confocal microscope was employed to take immunostaining images. Antibodies used in this study: mouse anti Ptc (1:100, DSHB), rat anti Ci (1:100, 2A1, DSHB), mouse anti Ubx (1:100, DSHB), mouse anti Smo (1:200, DSHB), rabbit anti Pc (1:200, Santa Cruz), mouse anti Flag (1:500, Invitrogen), mouse anti V5 (1:500, Sigma), mouse anti HA (1:500, Sigma), rabbit anti Yki (1:100, a gift from Dr Lei Zhang, SIBCB). Secondary antibodies conjugated to A488, Cy3 or Cy5 (Jackson ImmunoResearch) were used at 1:1000.

Induction of gene RNAi or overexpression

The gene RNAi and overexpression using the *tubGal80^{ts}; ciGal4* were performed in the Gal4-Gal80^{ts} system[6]. All flies were raised in 18 °C before the third instar larvae stage, and the third instar larvae were then shifted from 18 °C to 29 °C for 24 hrs, at which temperature Gal80^{ts} is inactivated and Gal4 is permitted to drive the gene expression. After this, the flies were returned back to 18 °C until eclosion. If we detected the exogenous RNA level or the exogenous protein level, we would dissect the larvae after the induction in 29 °C immediately. The adult wings were analyzed at the 3 days after eclosion. The photos were taken using the Lecia S8APO. All controls were crossed with the yw flies. While the *ptcGal4*, *dppGal4* and *hhGal4* used here adopted the Gal4 systems under the normal systems in RT unless other statements.

Total RNA extraction and RT-qPCR

Wing discs with indicated genotypes were lysed in Trizol Reagent (Invitrogen) directly after dissected from the larvae for total RNA extraction following the standard protocol. For RT-qPCR, 0.5 μg total RNA was used for reverse transcription with ReverTra Ace qPCR RT Master Mix with gDNA Remover (FSQ-301, Toyobo). Real time qPCR was conducted on the LightCycler 96 Real time PCR system (Roche) with SYBR Green Realtime PCR Master Mix (QPK-201, Toyobo). The $2^{-\Delta\Delta Ct}$ method was used for quantification. The primer pair used are listed as follows:

drl: AATGTGCACTCTGGGTAAACTG; ATAATGGTGAAGAGCTCATCGG
tub84b: GAGTCAGACCTCGAAATCGT; TGGATAGAGATACATTCACGCA
cnc: CATCGTCATGTCTTCCAGTACC; TTCACGTGGTAGCAAGTAGAC
hsp23: CTTCATCACTCGTCACTTTGTC; TCCTCTACTTATCGTTGCCA
antp: GTTTGGTAAGTGTCAAGAACGC; GGGTCAAGTAGCGATTGAAGTG
rho: TGTCATCTTTGTCTCCTGCGA; AAAGCCGATCGTTAGTCCTG
sc: GCACATAATGCCAGCCCCTA; GAGCATCTGGTCAGTGCCAT
hbs: TCGGAGAACGTTTACAAGTTCAC; CTTGGGAGCATACAGGACAG
s6k: CTAGTTTATGCCTTCCAGACAGAC; TCGCTTAAATAGAAGCATGTGG
rac1: AGGCTAAATGGCTCATCCGT; ACAGAACACTCGAAACAAACTCAG
spi: TCCAGTCACTAGTAGCACCAC; GCATCGTTCAAACAGTACCAG
ht1b: GAGAAACTTGCAGAATGTTGCC; ATCCATTACTTATCTCGTAGACGG
hep: ATCAAGTGCCTCACAAAGAACC; GTTATCCTTGATGCTCTGAAACCA
actin5c: CCAGCAGTCGTCTAATCCAG; CAGCAACTTCTTCGTCACAC
fwe: AATAGCCATGTCGTTTGCGG; AAGAATGGCAAAGAAGGCGG
tkv: TCCAACAGCTTTACGATGAGAC; CAGCAGACAATGTTCTTGCC
lola: GATACGTCTGGCGATGTGTC; TTGTCGTGGGCATCATTCTC
ulp1: GAACAGCAGGCTAATGAAAGCA; CTTGGTCTCTTTCTTCTCTTCCG
en: CTTAAGCGGGAGTTCAACGAG; CCGTCGACTTCTTGATCTTGG

dpp: GGCTTCTACTCCTCGCAGTG; TAATGCTGTGCTGGTCGAGG

pc: GGGAATTTACAAACTCCTGCT; ATGTACAATCAATACCGGCA

fs(1)h: CCGCCAAGAAAGATGAGTCC; TAACCTGCTTCACTGTCGCT

e2f1: TATTACAAGGTCAAACGTCGGC; CGTATGCTTCTGATACACCGTC

Robustness of trajectories for gene modular network

To examine robustness of the GMN in the wing disc development, we further analyzed the dynamic properties of the constructed network via the state-transition trajectories for all possible states[8-10]. Trajectory analysis showed that there are three different state-transition trajectories in the direction of A to P compartment. Among these three state-transition trajectories, the biggest or major one ends up with the attractor S_N = 000000000111 (the blue path, Fig. 4b), which is concordant with experimental data. We then made statistics on the distribution of each trajectory in this biggest trajectory. For 12 modules, we have 4,096 possible states in total. For each state, there is a trajectory from this initial state to the corresponding attractor. Thus, there are 4,096 possible trajectories in all. For each trajectory , we counted the number of common states with the major trajectory, if there are c common states, then the overlap ratio is $\frac{c}{13}$ (the major trajectory has 13 states in all). For each trajectory we can calculate a ratio, and the possible values of ratios are in set $\{\frac{0}{13},\frac{1}{13},\frac{2}{13},\cdots,\frac{13}{13}\}$, where 0 represents no overlap states. Hence, for each value in $\{\frac{0}{13},\frac{1}{13},\frac{2}{13},\cdots,\frac{13}{13}\}$, we can obtain a number N_i, i=0,1,……,13, with $\sum_i N_i = 4096$. Finally, we let

$$\text{Proportion}_i = p(\text{overlap ratio larger than } \frac{i}{13}) = \frac{4096 - N_i}{4096} \quad [S1]$$

where i=0,1,……,13. Then, we plotted the distribution of states overlap ratios against the proportions for the original GMN. We found that over half proportion of all paths have 8/13 overlap ratio with the major trajectory (Fig. 4c, red line plus dash line). In addition, we made random perturbations on state-transition table of the GMN in the original spatial diffusion table (Table S2), Specifically, we randomly flipped two bits in the original diffusion table (Table S2) and calculated attractors and transition tree for the perturbed GMN, we did this perturbation 1000 times. This kind of perturbations will not alter the structure of the Boolean network but may change the trajectory. We found that 99.92% of the 1000 perturbed GMN has the same attractors as the original GMN, which were 000000000111 (3658 states on average, 89.31%), 000000000000 (420 states on average, 10.25%) and 000110001000 (18 states on average, 0.44%) which reveals the robustness of the GMN for the major trajectory[8,11]. We also computed the distribution of states overlap ratios between all the other possible trajectories and the major trajectory for the perturbed network by Eq.[S1] (Fig. 4c, green line) in the same way. It has almost the same distribution of the states overlap ratios as the original GMN (Fig. 4c, red line vs green line).

Computational analysis of the GMN robustness in response to Hh production changes

The pathway enrichment analysis results showed that M8 is significantly enriched in Hedgehog signaling pathway (Fig. 2d in the main text). From this point of view, we believe that the variation of the state of M8 reflects

the change of Hh concentration level. In the physiological state, the state of M8 is 00000011110000 from A to P compartment, where 1 means the module is active. We assumed that the Hh concentration increases with the increase of the number of state 1 in M8 from A-P boundary to A compartment compared to M8 in the physiological state. And similarly, the decrease of Hh concentration leads to the decrease of the number of state 1 in M8 from A compartment to A-P boundary in comparison to M8 in the physiological state. Hence, we first replaced the original state of M8 by 00000111110000 in the spatial diffusion table (Fig. 5b in the main text). Then we reconstructed the new GMN and calculated attractors for the new GMN after perturbation by using the R package BoolNet[12]. We did the same things for the other cases as well, namely 00001111110000, 00011111110000, 00111111110000, and 01111111110000 in which case the concentration of Hh increasing step by step. On the other hand, we replaced the original state of M8 in the spatial diffusion table by 00000011100000, and we also reconstructed the new GMN and calculated its attractors. Similarly, we computed the cases 00000000110000 and 00000000010000 in which case the concentration of Hh decreases gradually.

References for Supplementary methods

1 Zhang, Z. *et al.* Ter94 ATPase complex targets k11-linked ubiquitinated ci to proteasomes for partial degradation. *Developmental cell* **25**, 636-644, doi:10.1016/j.devcel.2013.05.006 (2013).

2 Zhao, Y., Tong, C. & Jiang, J. Hedgehog regulates smoothened activity by inducing a conformational switch. *Nature* **450**, 252-258, doi:10.1038/nature06225 (2007).

3 Yang, X. *et al.* Drosophila Vps36 regulates Smo trafficking in Hedgehog signaling. *J Cell Sci* **126**, 4230-4238, doi:10.1242/jcs.128603 (2013).

4 Zhang, Z., Lv, X., Jiang, J., Zhang, L. & Zhao, Y. Dual roles of Hh signaling in the regulation of somatic stem cell self-renewal and germline stem cell maintenance in Drosophila testis. *Cell research* **23**, 573-576, doi:10.1038/cr.2013.29 (2013).

5 Pan, C. *et al.* UbcD1 regulates Hedgehog signaling by directly modulating Ci ubiquitination and processing. *EMBO Rep* **18**, 1922-1934, doi:10.15252/embr.201643289 (2017).

6 Zhang, S. *et al.* Repression of Abd-B by Polycomb is critical for cell identity maintenance in adult Drosophila testis. *Scientific reports* **7**, 5101, doi:10.1038/s41598-017-05359-0 (2017).

7 Fan, J., Liu, Y. & Jia, J. Hh-induced Smoothened conformational switch is mediated by differential phosphorylation at its C-terminal tail in a dose- and position-dependent manner. *Dev Biol* **366**, 172-184, doi:10.1016/j.ydbio.2012.04.007 (2012).

8 Wang, G. *et al.* Process-based network decomposition reveals backbone motif structure. *Proceedings of the National Academy of Sciences of the United States of America* **107**, 10478-10483, doi:10.1073/pnas.0914180107 (2010).

9 Bornholdt, S. Boolean network models of cellular regulation: prospects and limitations. *J R Soc Interface* **5** **Suppl** **1**, S85-94, doi:10.1098/rsif.2008.0132.focus (2008).

10 Li, F., Long, T., Lu, Y., Ouyang, Q. & Tang, C. The yeast cell-cycle network is robustly designed. *Proceedings of the National Academy of Sciences of the United States of America* **101**, 4781-4786, doi:10.1073/pnas.0305937101 (2004).

11 Martin, S., Zhang, Z., Martino, A. & Faulon, J. L. Boolean dynamics of genetic regulatory networks inferred from microarray time series data. *Bioinformatics* **23**, 866-874, doi:10.1093/bioinformatics/btm021 (2007).

12 Mussel, C., Hopfensitz, M. & Kestler, H. A. BoolNet-an R package for generation, reconstruction and analysis of Boolean networks. *Bioinformatics* **26**, 1378-1380, doi:10.1093/bioinformatics/btq124 (2010).

Supplementary Figures and Tables

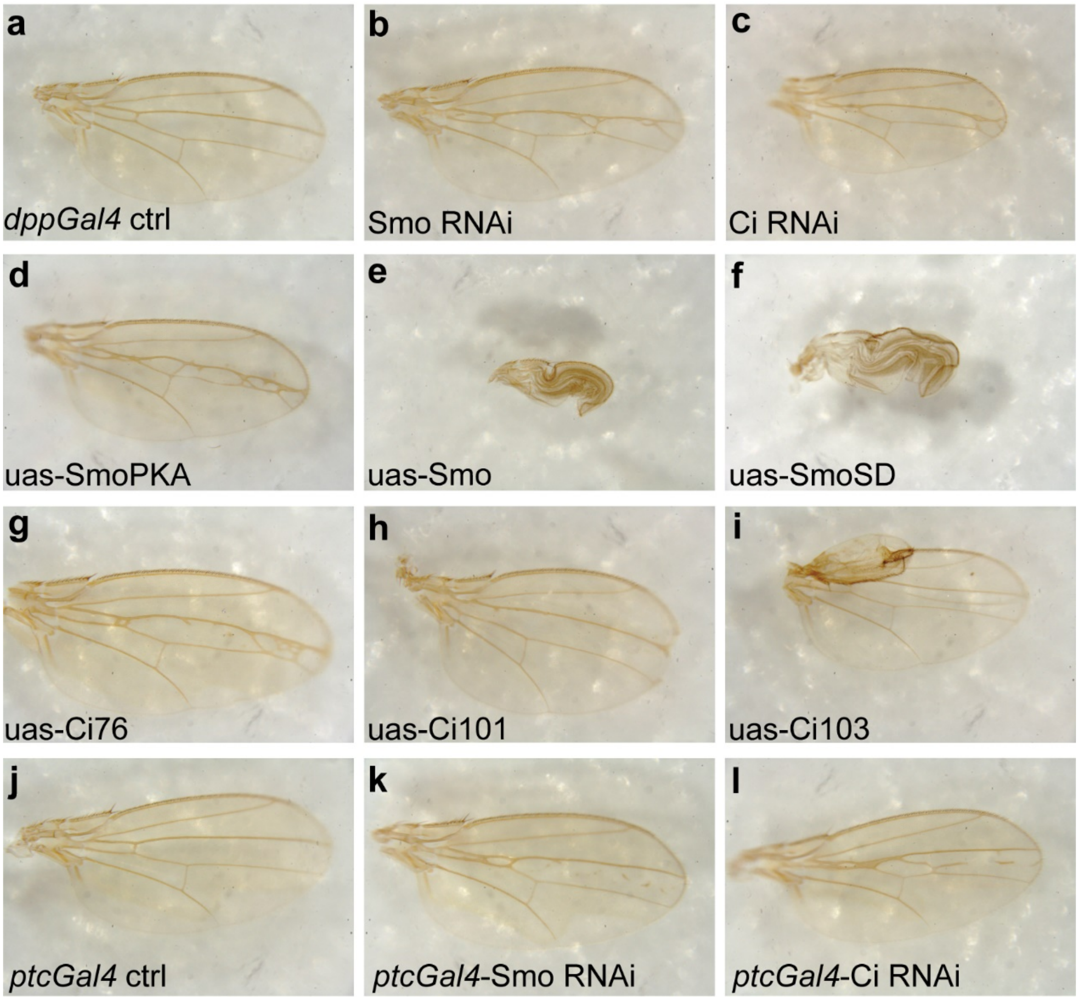

Supplementary Fig. S1 Hh signaling is critical for *Drosophila* wing development.

(a) An adult wing of *dppGal4* control. (b) An adult wing expressing Smo RNAi driven by *dppGal4* at room temperature. (c) An adult wing expressing Ci RNAi driven by *dppGal4* at room temperature. (d-f) Adult wing expressing different forms of Smo driven by *dppGal4*, (d) uas-SmoPKA (Smo dominant negative form), (e) uas-SmoWT (Smo wild type form), (f) uas-SmoSD (Smo constitutively active form) in 18 °C. (g-i) Adult wing expressing different forms of Ci driven by *dppGal4*, (g) uas-Ci76 (Ci

repressor form), (h) uas-Ci101 (Ci wild type form), (i) uas-Ci103 (Ci active form). (j-l) Adult wings driven by *ptcGal4*, (j) control, (k) Smo RNAi, (l) Ci RNAi.

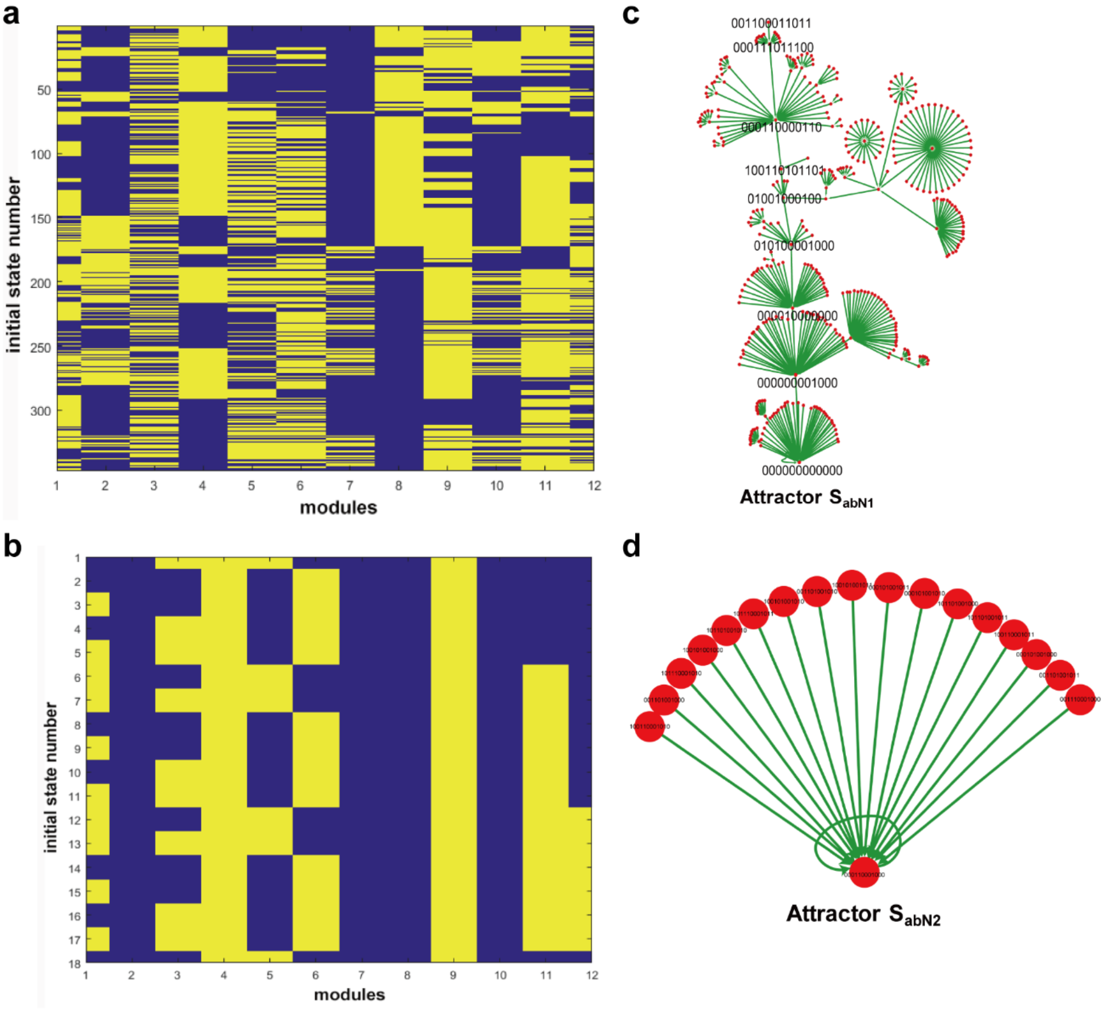

Supplementary Fig. S2 The abnormal attractors in the gene modular network.

(a) The clustering analysis of the initial states leading to S_{abN1}. (b) The clustering analysis of the initial states leading to S_{abN2}. (c) The state-transition trees leading to attractor S_{abN1}. (d) The state-transition trees leading to attractor S_{abN2}.

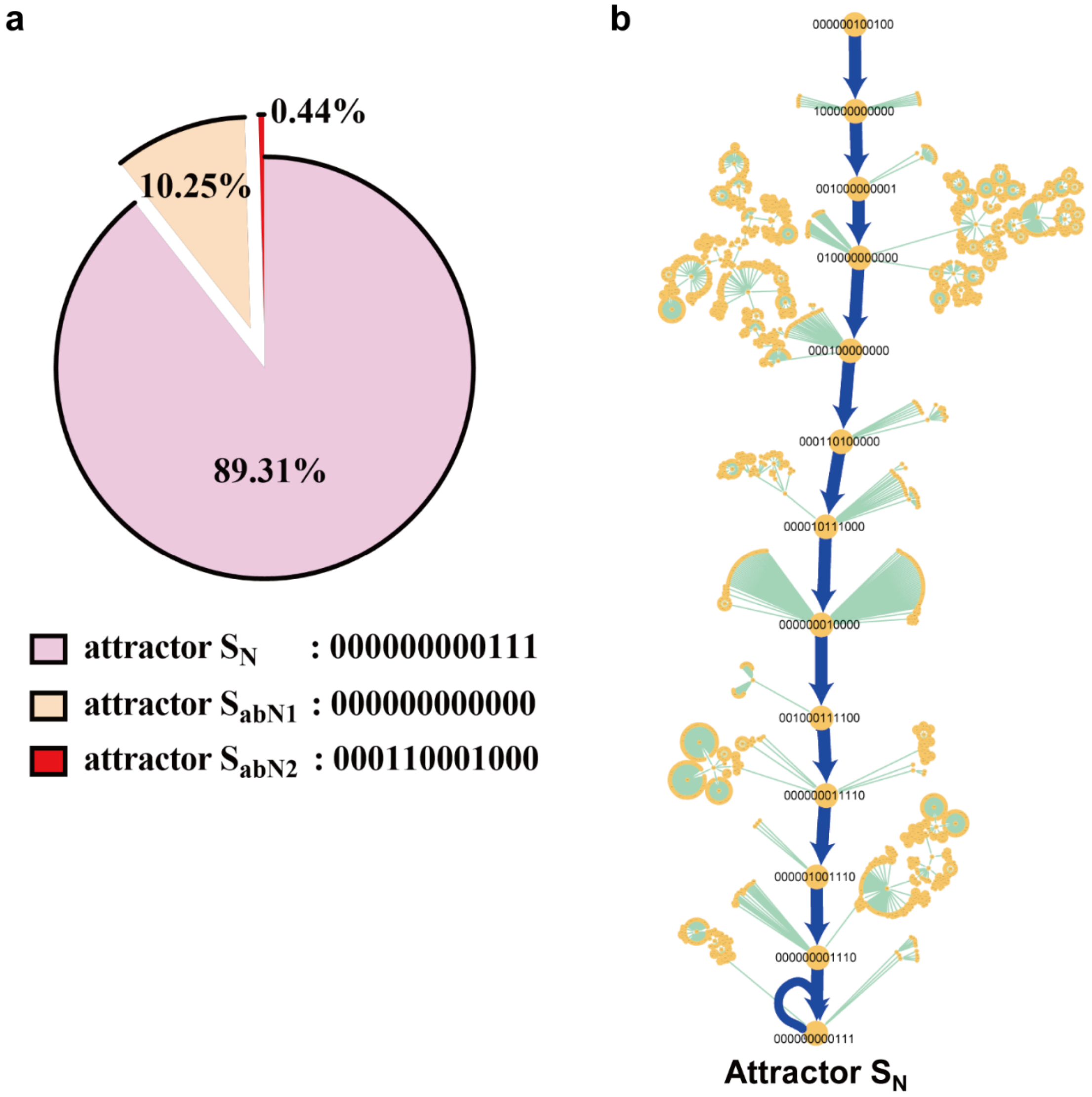

Supplementary Fig. S3 The attractors and the state-transition tree of the GMN after network perturbations.

We generated the original state transition table (Table S2) which is the original GMN, and then randomly perturbed one of the state transitions on the table. Based on the perturbed new state transition table or the new GMN, we calculated attractors and drew the state-transition tree. (a) The attractors of GMN after perturbation. There are three attractors: attractor normal (S_N,

90.14%), attractor abnormal 1 (S_{abN1}, 9.42%) and attractor abnormal 2 (S_{abN2}, 0.44%), which are the same as the original three attractors. (b) The biggest state-transition tree also converged to the attractor S_N. The major (physiological) trajectory (path) is labeled with navy lines. These clearly show the robustness of the GMN for both attractors and trajectories.

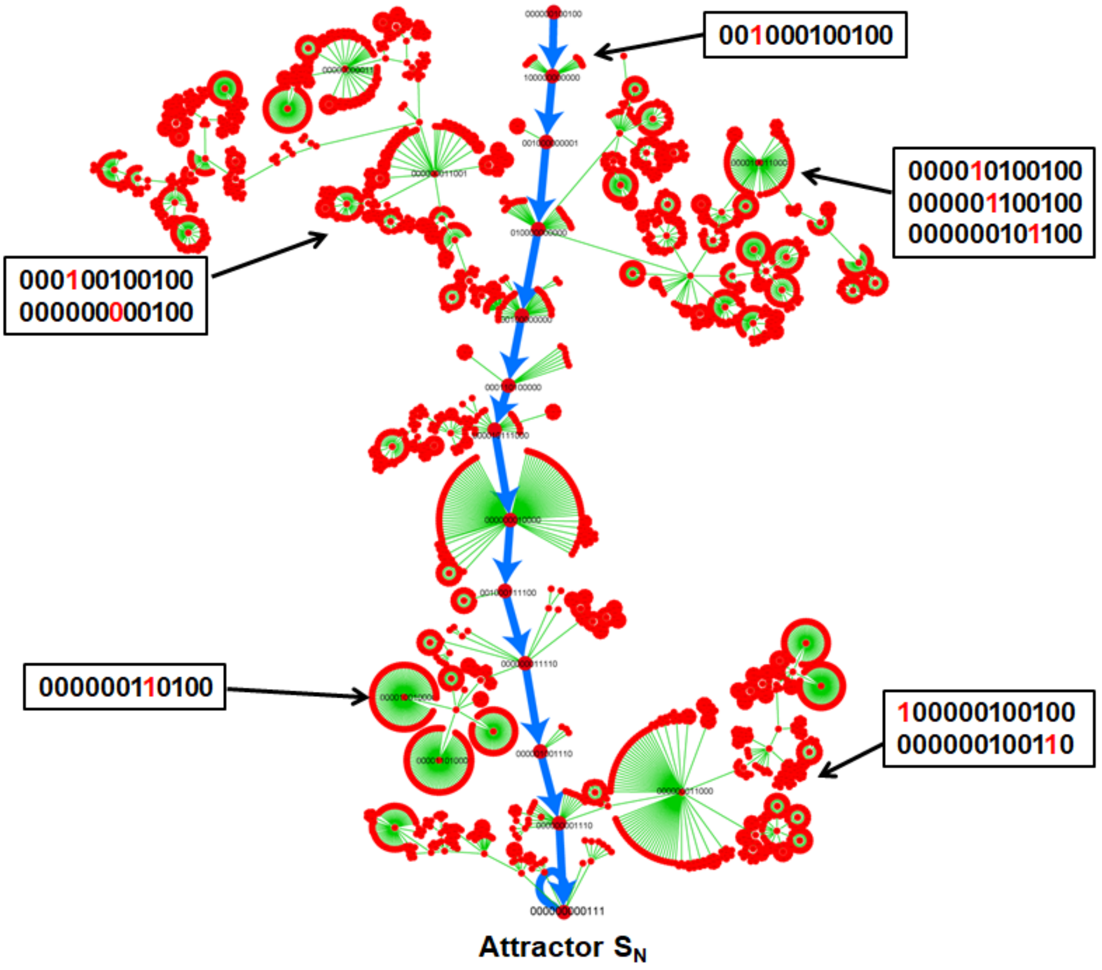

Supplementary Fig. S4 The modules in different initial states used to test the GMN robustness.

Here we chose these initial states which could go back to the attractor S_N in the end. These initial states only have one different module from the physiological initial state 000000100100. The red number indicates the changed module compared with physiological initial state 000000100100.

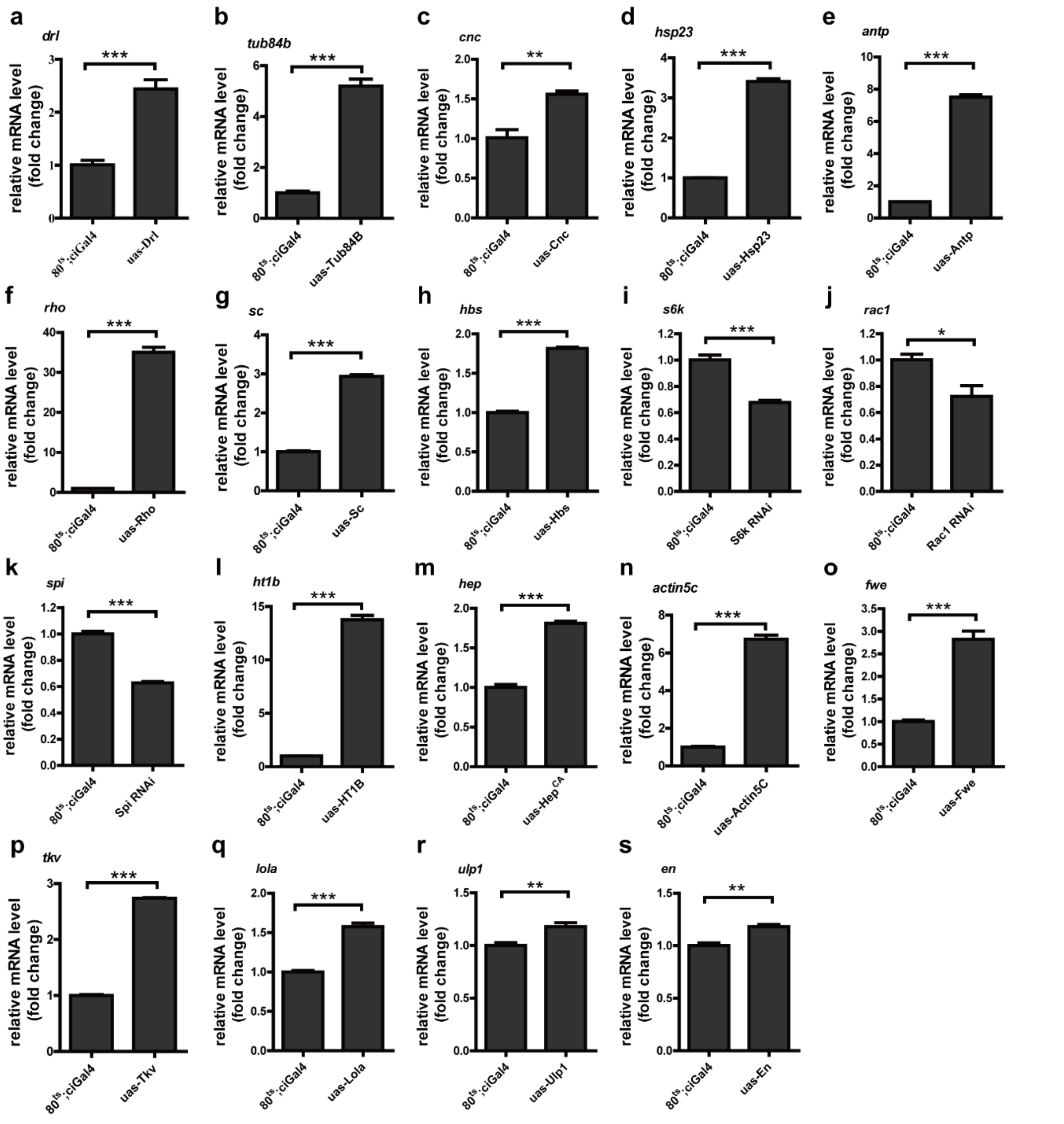

Supplementary Fig. S5 Transcriptional level changes detected by RT-qPCR in overexpression or RNAi flies.

The horizontal ordinates indicate the fly genotypes, *tubGal80^{ts}; ciGal4* control and the exogenous genes driven by *tubGal80^{ts}; ciGal4*. The Y-axis shows the relative mRNA level and the detected gene is listed over the Y-axis. Data is represented as mean±SEM, *p<0.05, **p<0.01, ***p<0.001, unpaired, one-tailed student's t test.

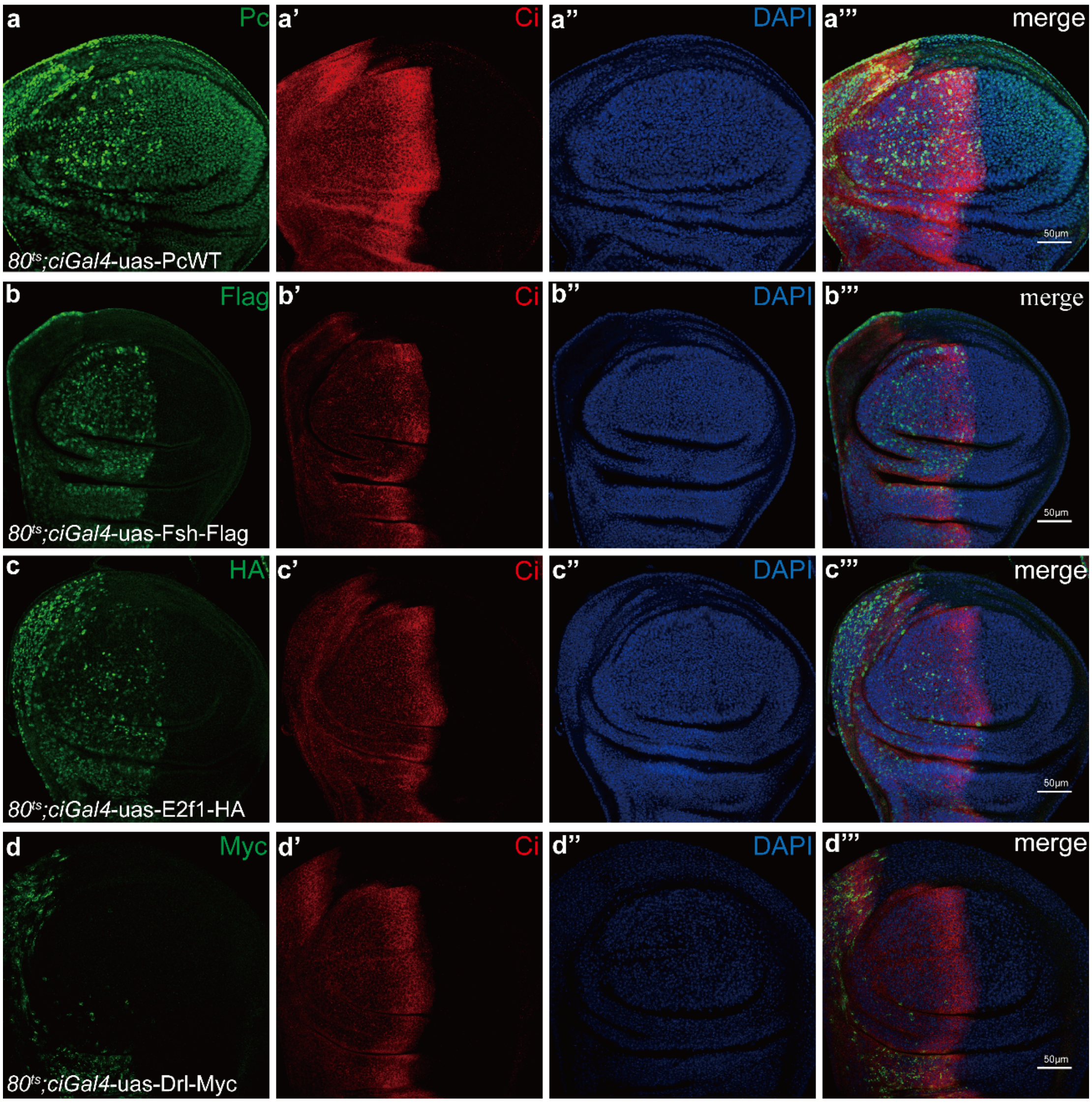

Supplementary Fig. S6 The decetion of exogenous protein expression level by immunostaining.

(a-a''') A representative *tubGal80^{ts}; ciGal4*-uas-PcWT wing disc was immunostained to show the expression level of Pc (green), Ci (red) and DAPI (blue) after the induction of PcWT at third instar larval stage for 24 hrs. Scale bar, 50 µm. (b-b''') A representative *tubGal80^{ts}; ciGal4*-uas-Fsh-Flag wing disc was immunostained to show the expression level of Flag (green), Ci (red) and DAPI (blue) after the induction of Fsh-Flag at third

instar larval stage for 24 hrs. Scale bar, 50 µm. (c-c''') A representative *tubGal80*^{ts}*; ciGal4*-uas-E2f1-HA wing disc was immunostained to show the expression level of HA (green), Ci (red) and DAPI (blue) after the induction of E2f1-HA at third instar larval stage for 24 hrs. Scale bar, 50 µm. (d-d''') A representative *tubGal80*^{ts}*; ciGal4*-uas-Drl-Myc wing disc was immunostained to show the expression level of Myc (green), Ci (red) and DAPI (blue) after the induction of Drl-Myc at third instar larval stage for 24 hrs. Scale bar, 50 µm.

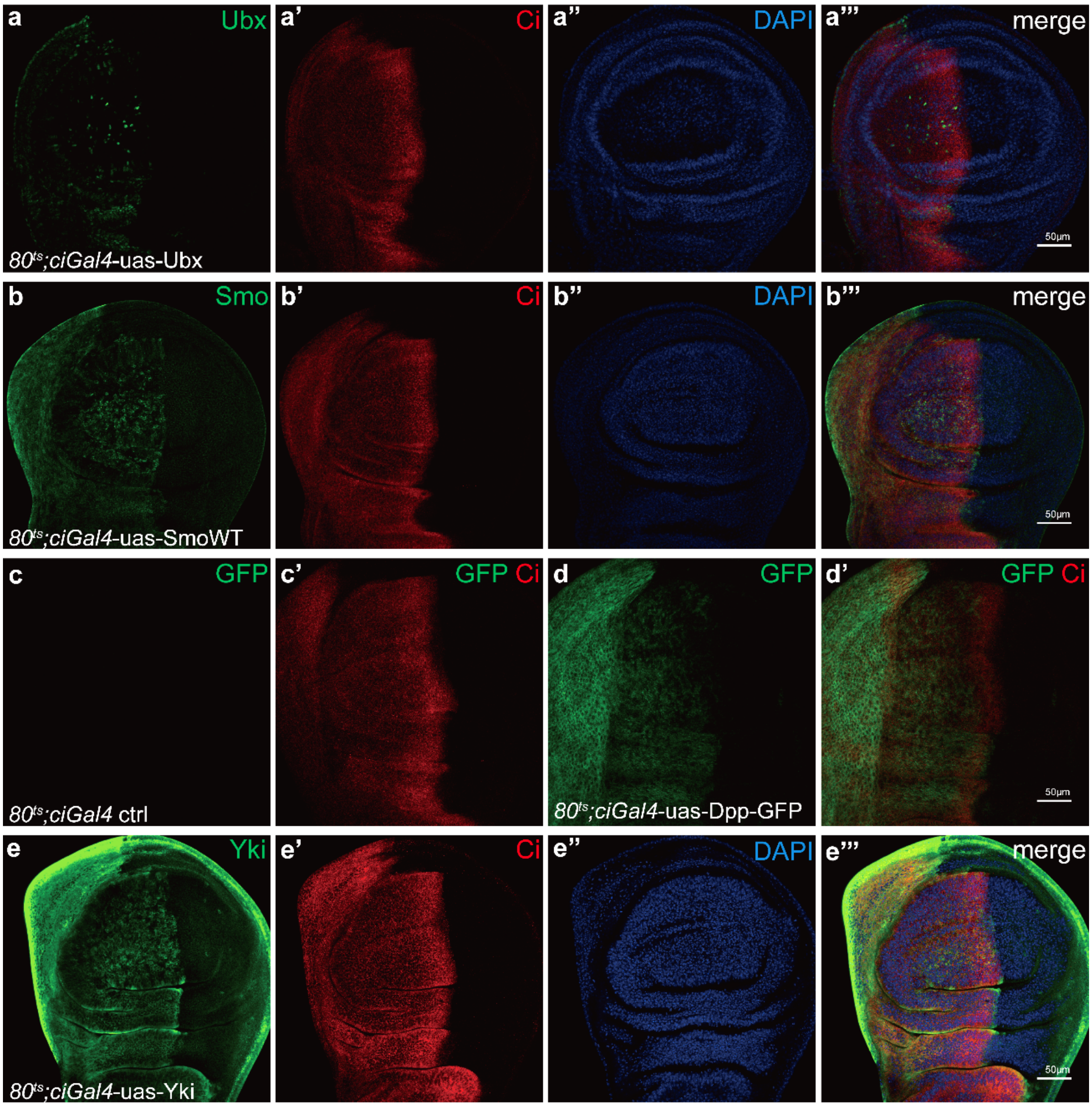

Supplementary Fig. S7 The protein expression level after the induction of exogenous Ubx, Smo, Dpp and Yki.

(a-a''') A representative *tubGal80*^{ts}*; ciGal4*-uas-Ubx wing disc was immunostained to show the expression level of Ubx (green), Ci (red) and DAPI (blue) after the induction of Ubx at third instar larval stage for 24 hrs. Scale bar, 50 μm. (b-b''') A representative *tubGal80*^{ts}*; ciGal4*-uas-SmoWT wing disc was immunostained to show the expression level of Smo (green),

Ci (red) and DAPI (blue) after the induction of SmoWT at third instar larval stage for 24 hrs. Scale bar, 50 µm. (c-d') The representative immunostained wing discs of *tubGal80^{ts}; ciGal4* control and *tubGal80^{ts}; ciGal4*-uas-Dpp-GFP, uas-Dpp-GFP is specifically induced at third instar larval stage for 24hrs. (C-C') *tubGal80^{ts}; ciGal4* control, (D-D') *tubGal80^{ts}; ciGal4*-uas-Dpp-GFP. GFP (green), Ci (red), DAPI (blue). Scale bar, 50 µm. (e-e''') A representative *tubGal80^{ts}; ciGal4*-uas-Yki wing disc was immunostained to show the expression level of Yki (green), Ci (red) and DAPI (blue) after the induction of Yki at third instar larval stage for 24 hrs. Scale bar, 50 µm.

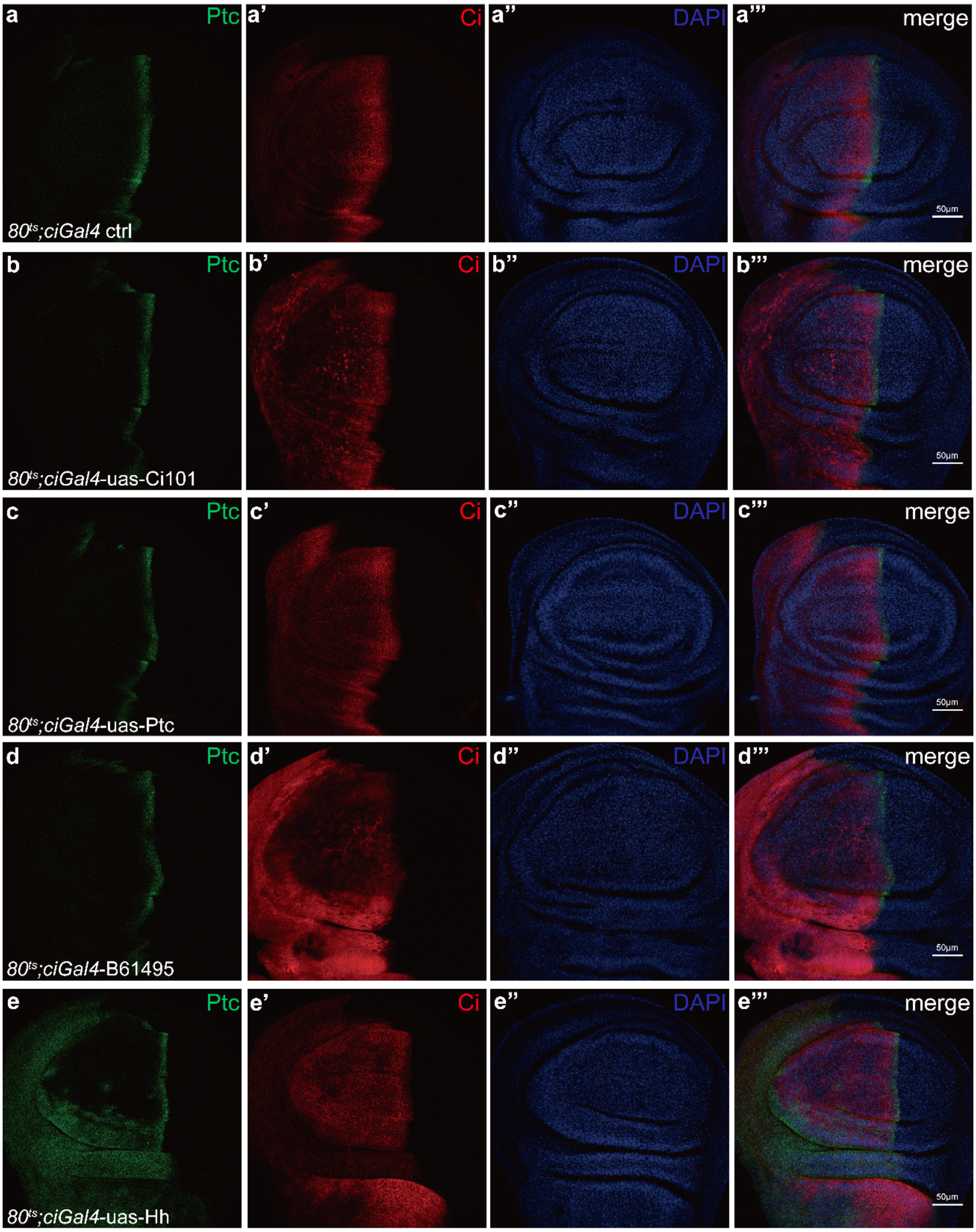

Supplementary Fig. S8 The protein expression level detection after the induction of exogenous Ci, Ptc, mir-1012 and Hh.

(a-a''') A representative *tubGal80*^{ts}*; ciGal4* control wing disc was immunostained to show the expression level of Ptc (green), Ci (red) and DAPI (blue). Scale bar, 50 μm. (b-b''') A representative *tubGal80*^{ts}*; ciGal4*-uas-Ci101 wing disc was immunostained to show the expression level of Ptc (green), Ci (red) and DAPI (blue) after the induction of Ci101 at third instar larval stage for 24 hrs. Scale bar, 50 μm. (c-c''') A representative *tubGal80*^{ts}*; ciGal4*-uas-Ptc wing disc was immunostained to show the expression level of Ptc (green), Ci (red) and DAPI (blue) after the induction of Ptc at third instar larval stage for 24 hrs. Scale bar, 50 μm. (d-d''') A representative *tubGal80*^{ts}*; ciGal4*-uas-mir-1012 (B61495) wing disc was immunostained to show the expression level of Ptc (green), Ci (red) and DAPI (blue) after the induction of mir-1012 at third instar larval stage for 24 hrs. There is a significant change of Ci expression pattern indicating the function of mir-1012 overexpression. Scale bar, 50 μm. (e-e''') A representative *tubGal80*^{ts}*; ciGal4*-uas-Hh wing disc was immunostained to show the expression level of Ptc (green), Ci (red) and DAPI (blue) after the induction of Hh at third instar larval stage for 24 hrs. Scale bar, 50 μm.

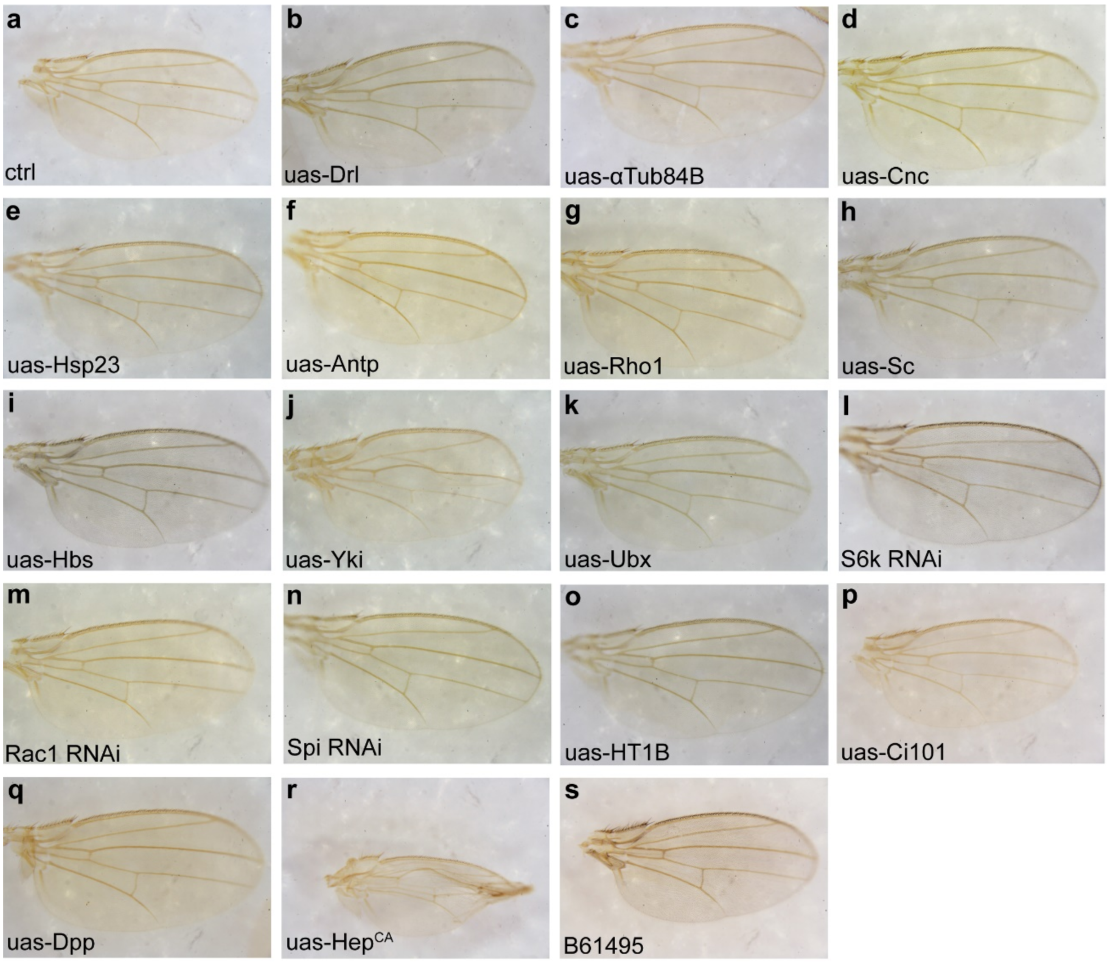

Supplementary Fig. S9 Adult wing phenotypes after the induction of exogenous genes.

Adult wings driven by the *tubGal80^{ts}*; *ciGal4*, in which genes are specifically expressed in the third instar larvae stage in A compartment, are used to mimic the change of the initial states.

(a) A representative adult wing picture of *tubGal80^{ts}; ciGal4* control. (b-s) The representative adult wing pictures after the induction of the given genes. (b-c) The genes come from M1, the wings are normal. (d) The gene comes from M3, the wing is normal. (e-i) The genes come from M4 with normal

wings. (j-k) The genes come from M5, the wing of *tubGal80*^{ts}*; ciGal4*-uas-Yki is abnormal. (l-n) The genes come from M7, the wings are normal. (o-s) The genes come from M8, the wing of *tubGal80*^{ts}*; ciGal4*-uas-Hep^{CA} is abnormal.

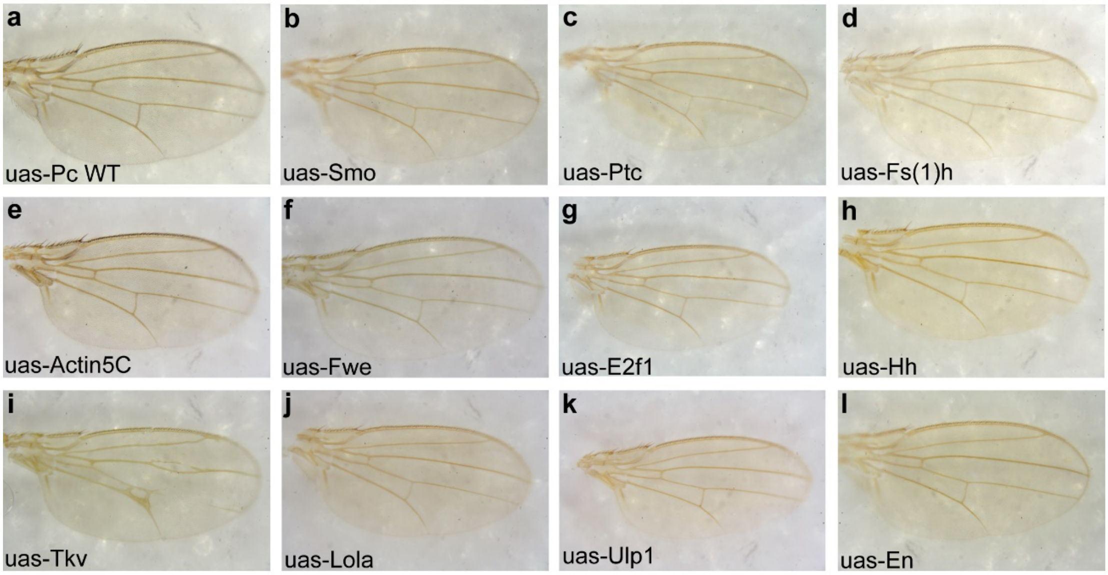

m

module	M1	M3	M4	M5	M7	M8	M9	M11	total
number of normal wings	2	1	5	1	3	4	6	5	27 (90%)
number of abnormal wings	0	0	0	1	0	1	0	1	3 (10%)
total	2	1	5	2	3	5	6	6	30 (100%)

Supplementary Fig. S10 Adult wing phenotypes after the induction of exogenous genes (continued).

(a-f) The genes come from M9, and the wings are normal. (g-l) The genes come from M11, and the wing of *tubGal80*^{ts}; *ciGal4*-uas-Tkv is abnormal. (m) The statistical table of the adult wing phenotype analysis. There are only three abnormal wings from M5, M8 and M11. The proportion of the abnormality is 10%.

Supplementary Table S1 Spatial diffusion of the binary states

	A1	B1	A2	B2	B3	A3	B4	A4	B5	A5	B6	A6	B7
**M1**	0	1	0	0	0	0	0	0	0	0	0	0	0
**M2**	0	0	0	1	0	0	0	0	0	0	0	0	0
**M3**	0	0	1	0	0	0	0	0	1	0	0	0	0
**M4**	0	0	0	0	1	1	0	0	0	0	0	0	0
**M5**	0	0	0	0	0	1	1	0	0	0	0	0	0
**M6**	0	0	0	0	0	0	0	0	0	0	1	0	0
**M7**	1	0	0	0	0	1	1	0	1	0	0	0	0
**M8**	0	0	0	0	0	0	1	1	1	1	0	0	0
**M9**	0	0	0	0	0	0	1	0	1	1	1	1	0
**M10**	1	0	0	0	0	0	0	0	1	1	1	1	1
**M11**	0	0	0	0	0	0	0	0	0	1	1	1	1
**M12**	0	0	1	0	0	0	0	0	0	0	0	0	1

Supplementary Table S2 Spatial diffusion of the binary states in theory

	A1	B1	A2	B2	B3	A3	B4	A4	B5	A5	B6	A6	B7	B7*
**M1**	0	1	0	0	0	0	0	0	0	0	0	0	0	0
**M2**	0	0	0	1	0	0	0	0	0	0	0	0	0	0
**M3**	0	0	1	0	0	0	0	0	1	0	0	0	0	0
**M4**	0	0	0	0	1	1	0	0	0	0	0	0	0	0
**M5**	0	0	0	0	0	1	1	0	0	0	0	0	0	0
**M6**	0	0	0	0	0	0	0	0	0	0	1	0	0	0
**M7**	1	0	0	0	0	1	1	0	1	0	0	0	0	0
**M8**	0	0	0	0	0	0	1	1	1	1	0	0	0	0
**M9**	0	0	0	0	0	0	1	0	1	1	1	1	0	0
**M10**	1	0	0	0	0	0	0	0	1	1	1	1	1	1
**M11**	0	0	0	0	0	0	0	0	0	1	1	1	1	1
**M12**	0	0	1	0	0	0	0	0	0	0	0	0	1	1

Supplementary Table S3 Regulation rules for the GMN (Boolean functions).

Module	Gene Modular network regulation rules
**M1**	NOT M9 AND M10 AND NOT M12
**M2**	NOT M11 AND M12
**M3**	NOT M1 AND M8 AND NOT M9<br>OR M1 AND NOT M8 AND NOT M9
**M4**	NOT M2 AND M4 AND NOT M7<br>OR M2 AND NOT M4 AND NOT M7
**M5**	M4
**M6**	M8 AND M11
**M7**	NOT M4 AND M8 AND NOT M9<br>OR M4 AND NOT M8 AND NOT M9
**M8**	M8 AND NOT M10<br>OR M7 AND NOT M10<br>OR M7 AND NOT M8
**M9**	NOT M5 AND NOT M6 AND M8<br>OR NOT M5 AND M6 AND NOT M8<br>OR M5 AND NOT M6 AND NOT M8
**M10**	NOT M7 AND M10<br>OR NOT M7 AND M8<br>OR M8 AND M10
**M11**	NOT M9 AND M10 AND M12<br>OR M9 AND M10 AND NOT M12
**M12**	NOT M1 AND NOT M6 AND NOT M8 AND M11<br>OR M1 AND NOT M6 AND NOT M8 AND NOT M11

Supplementary Table S4 The description on mathematical terms in the Boolean network/system (Eq.[1])

Terms	Description	Implication
**Attractor**	Steady state/states toward which a system or network tends to evolve, by Eq.[1].	A gene/protein modular profile/state at the end of a biological process is considered as an attractor
**Normal attractor**	Attractor which is concordant with physiological data	This gene modular profile/state is in the P compartment in wing disc, which is the end of wing disc development
**Abnormal attractor**	Attractor which is not concordant with physiological data	This gene modular profile/state does not correspond to normal physiological data
**Trajectory**	A path with a series of states for which a system/network follows from an initial state, by Eq.[1]	A trajectory of the GMN corresponds to a wing disc developmental path, which is also a path of states from an initial state (or gene profile) to an attractor
**Major trajectory**	The trajectory with states which are all consistent with physiological data	The trajectory corresponds to the path of the normal wing disc development
**Basin of an attractor**	All of those states which lead to this attractor, by Eq.[1]	It is composed of all gene modular states which converge to this attractor
**State-transition tree of an attractor**	All of those trajectories which lead to this attractor	It is made of those trajectories or state-path which converge to this attractor
